# Understanding equity and diversity needs among health library professionals in Canada: a survey

**DOI:** 10.29173/jchla29700

**Published:** 2024-04-01

**Authors:** Abiola Ajayi, Patricia L. Foster, Chau Ha, Maria Zych, Tara Landry

**Affiliations:** 1 Lead Librarian, Sunnybrook Health Sciences Centre, Toronto, ON; 2 UBC Woodward Library, Integrated Sciences, Vancouver, BC; 3 Liaison and Instruction Librarian, Saskatchewan Polytechnic Library, Saskatoon Campus, Saskatoon, SK; 4 Liaison and Instruction Librarian, Dentistry Library, University of Toronto, Toronto, ON; 5 Head of Reference and Collection Development, Bibliothèque de la santé, Université de Montréal, Montréal, QC

## Introduction

The publication of the Truth and Reconciliation Commission’s final report in 2015 [1] has raised awareness of Equity, Diversity, and Inclusion (EDI) matters within many Canadian organizations. More recently, the increased incidence of racism following the COVID-19 pandemic [2-4], the murders of Americans George Floyd, Breonna Taylor, and Ahmaud Arbery [5], and the death of Joyce Echaquan [6] have sparked outrage in Canada. Systemic racism has been at the forefront of discussions in many of our institutions and communities, challenging our systems and leaders to rethink the status quo and push for a more inclusive, diverse and equitable environment.

Like other professionals, librarians responded to anti-racism movements by creating statements of support for ending systemic racism, and by implementing strategic goals to improve librarianship via local management, professional organizations, and national and international networks [7].

By summer 2020, it was evident that CHLA/ABSC needed to take action and make improvements in matters of EDI and social justice initiatives. As a first step, an EDI Task Force (EDITF) was formed in September 2020. Its purpose was “to assist the Association to move forward on matters of equity, diversity and inclusion, by examining the Association in its current state to determine areas for improvement” [8]. The EDITF assisted the Board with its operational plan in early 2021, and consulted on various position statements published by the Board in 2021 [9] and 2022 [10]. In 2021, planning began on a survey to gather demographic information about CHLA/ABSC members and their needs related to EDI.

Many professional associations have previously used surveys to gather feedback about EDI activities and future directions. The American Library Association (ALA) launched three surveys from January 2015 to March 2016 to gain feedback from current and potential members regarding the climate of ALA [11].

The Medical Library Association (MLA) appointed members to a Diversity and Inclusion Task Force (DITF) in 2017 to review the language in their strategic documents, such as their mission, vision, values, and ethical guidelines [12]. In addition, the DITF ran a survey in 2019 to collect members’ demographic information and assess how they felt about MLA's EDI initiatives [13].

In Canada, the Canadian Academic and Research Libraries (CARL) launched its own survey in October 2021 [14]. Its purpose was to gather demographic information, gauge employee feedback on current EDI initiatives, and establish guidelines to evaluate and measure the impact of CARL libraries’ strategies and practices with respect to diversity and inclusion [14].

Finally, the Visible Minority Librarians of Canada (ViMLoC) updated their 2013 EDI survey and relaunched it in 2021 [15], gathering demographic, education, and employment information of visible minority librarians (VMLs) working in Canadian institutions.

In the Fall of 2021, CHLA/ABSC’s EDITF began working on developing its own survey with the purpose of: (i) gathering demographic information about CHLA/ABSC members and (ii) assessing the support, education, and leadership needs of Canadian health information professionals relating to diversity and inclusion.

## Description

The development of the survey was an iterative process. The EDI surveys described above, along with previous CHLA/ABSC member surveys [16], were consulted and adapted. New questions relevant to health information professionals in Canada were added. Questions related to CHLA/ABSC’s role in matters of EDI, as well as on continuing education (CE) topics of interest, were also included. These were designed to collect data on respondents’ priorities in order to improve the Association’s efforts moving forward.

In December 2022, two surveys, French and English, were created using SurveyMonkey [17]. The survey was reviewed and tested before distribution by the members of the EDITF. The survey consisted of 31 questions and used multiple-choice, rating scales, and open-ended questions (see Online Supplement Appendix 1).

Online Supplement Appendix

The final survey was distributed by email to CHLA/ABSC members in January 2023. Respondents were also invited to participate via the CANMEDLIB listserv and CHLA/ABSC Chapter Presidents’ listservs for distribution to Chapter members who may not be members of CHLA/ABSC. Links to the survey were posted on social media (Facebook, Instagram, Twitter) as well as on the CHLA/ABSC website. The survey ran from January 10 to January 31, 2023. All responses were collected anonymously and respondents had the option of skipping any question they did not wish to answer. A summary of the responses to all questions is available in Online Supplement Appendix 2.

Online Supplement Appendix

## Outcomes

### Association information (questions 1 & 2)

**Fig. 1:**
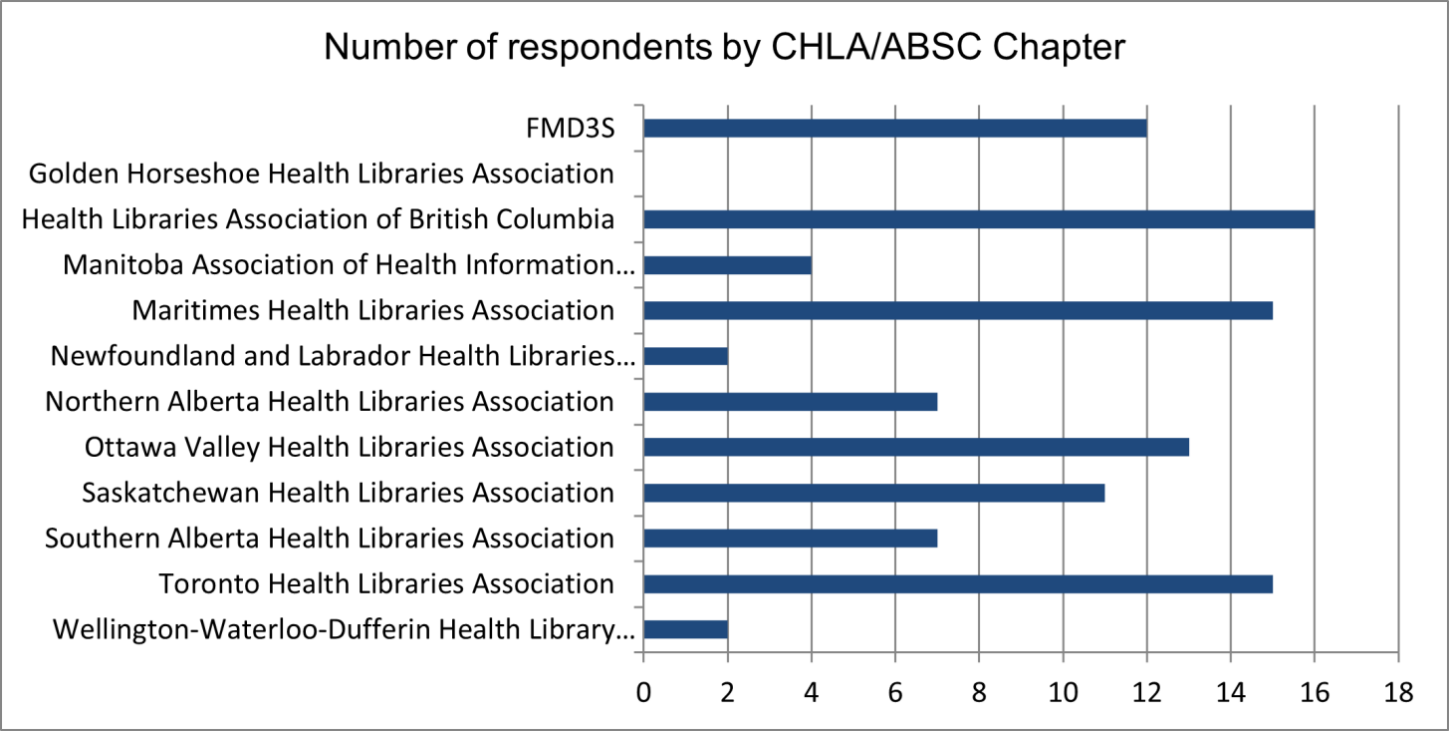
CHLA/ABSC chapters members

The first two questions of the survey sought to identify whether respondents were members of CHLA/ABSC or of a Chapter. The majority of respondents (83% or N=138) were CHLA/ABSC members, with 17% (N=28) of responses coming from non-members. Over 60% of respondents (62%, N=104) were also members of CHLA/ABSC Chapters. Figure 1 illustrates the breakdown of chapter membership among the respondents of the survey.

### Employment status (questions 3-11)

152 respondents (92%) indicated they worked in a library position, with 138 holding full-time permanent positions (91%). Most respondents held librarian positions (67%, N=101), followed by administrator or manager positions (16%, N=24) or library assistant/technicians and information specialist positions (8%, N=12 each). Two stated “Other” (one librarian and management, the other a program coordinator).

Question seven referred to library type. The majority of respondents indicated that they worked in a hospital setting (43%, N=65) or in a post-secondary institution (42%, N=64); followed by special libraries (13%, N=20); consortia (2%, N=3); a publisher (1%, N=2); and one person in a public library. Eight respondents (5%) said they worked in other libraries or organizations, with responses ranging as follows: notfor-profit, government, health research organization, healthcare outside of hospital, and two in public health.

Question nine asked about union membership. A little over half (52%, N=79) of respondents indicated that they were unionized, while the other half were not (47%, N=72).

As to the question of Academy of Health Information Professionals (AHIP) certification, while the majority of respondents were not currently certified (96%, N=159), 58% (N=91) said they were undecided or unsure if they would pursue AHIP certification in the future.

### Professional development and mentorship (questions 12-14)

Most respondents stated that their employers provided funds for professional development (84%, N=135), which they could either access via a personal fund that they could use at their discretion (42%, N=56), or via a group fund that they must obtain approval for (58%, N=77).

**Fig. 2:**
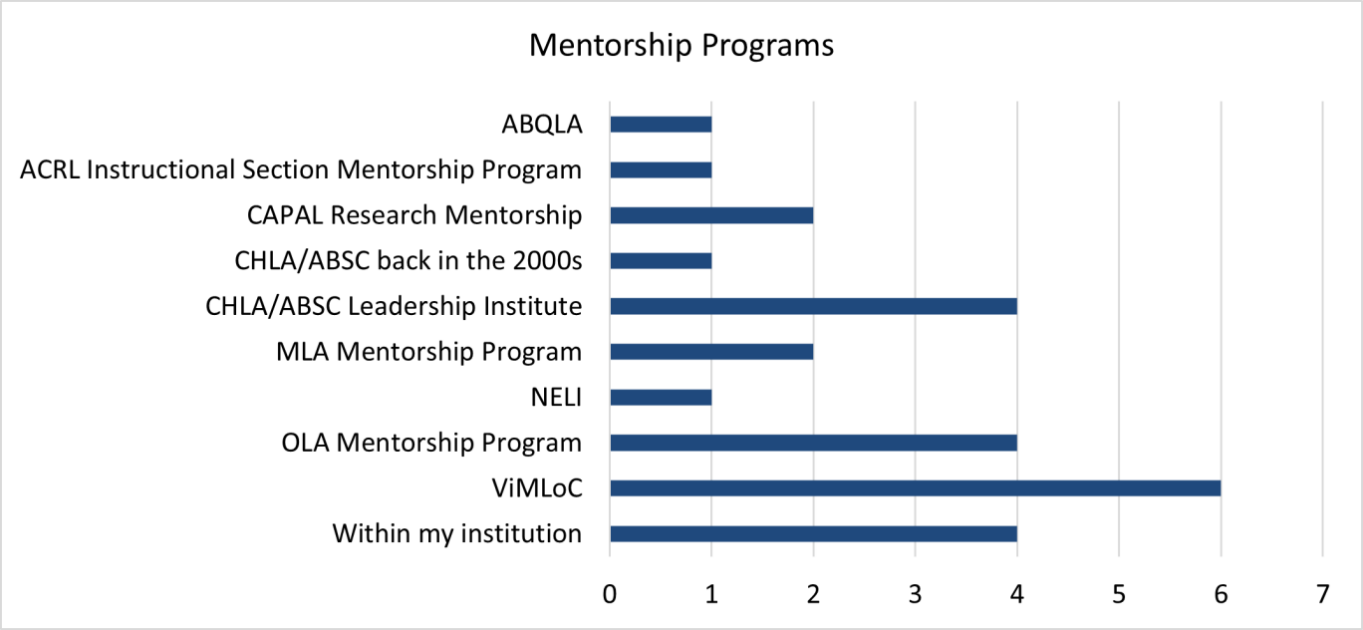
Mentorship programs mentioned by respondents

When asked whether they had participated in mentorship programs that they would recommend, 138 (87%) answered that they had not, while 21 responded they had and provided recommendations (see Figure 2 for details).

### Race and ethnicity (questions 15 and 16)

21 respondents (13%) indicated they considered themselves as belonging to a visible minority group, while 140 (86%) did not. The survey used ViMLoC’s definition of visible minority as Chinese, South Asian, Black, Filipino, Arab, West Asian, Southeast Asian, Latin American, Japanese, and Korean [15]. Five respondents (3%) identified as being of Indigenous ancestry, defined as whether a person has ancestry associated with First Nations, Métis, and/or Inuit [18].

### Ability (question 17)

The majority of respondents (72%, N=116) did not consider themselves as being a person with a disability, while 41 respondents (25%) reported having a disability, and four did not wish to respond (2%). Table 1 shows the self-identified disabilities from the respondents, who could select more than one from the list provided.

**Table 1:** Self-identified disabilities*

Disability	Percentage of responses	Number of responses
Sensory (e.g., Seeing, Hearing)	1%	2
Physical (e.g., Mobility, Flexibility, Dexterity, Chronic Pain)	8%	13
Cognitive (e.g., Learning, Developmental, Memory)	4%	7
Mental Health-Related (e.g., Depression, Anxiety, ADHD)	20%	32
Other/Unknown	3%	5
I do not consider myself to be a person with a disability	72%	116
I do not wish to respond	2%	4
**Answered**		161
**Skipped**		6

* Each respondent could select more than one option

### Sexual and gender identities (question 18)

20% of respondents (N=33) indicated that they identified as being part of the 2SLBGTQIA+ community, defined as: Two-Spirit, Lesbian, Gay, Bisexual, Transgender, Queer or Questioning, Intersex, Asexual, and additional sexual orientations and gender identities [19].

### Age (question 19)

The largest represented age group was 35-44 years (30%; N=48), followed by 45-54 years (29%; N=47), 25-34 years (20%, N=32), and 55-64 years (19%, N=30). The smallest group was 65+ (3%, N=5).

### Province (question 20)

Most of the respondents of the survey were from Ontario (43%, N=70); followed by Quebec (14%, N=22); British Columbia (12%, N=19); Alberta and Saskatchewan (both 8%, N=13); Nova Scotia (6%, N=11); New Brunswick (4%, N=6); and Newfoundland and Labrador (1%, N=2). Three said “Other”. Unfortunately, there were no respondents from Manitoba, the Northwest Territories, Nunavut, Prince Edward Island, or the Yukon.

### Work experience (question 21)

Years of experience working in libraries varied, with the most common being over 15 years (32%, N=52); followed by 11-15 years (23%, N=38); 6-10 years (21%, N=35); 1-5 years (19%, N=32); and less than one year (4%, N=6).

### Education (question 22)

88% of respondents (N=138) indicated that they had or were in the process of completing a Master’s degree, while two percent (N=4) had or were in the process of completing a PhD. The remaining ten percent indicated that they had college or university diplomas (N=11), certificates (N=1), or other post-secondary education degrees (N=4).

### Salary range (question 23)

**Fig. 3:**
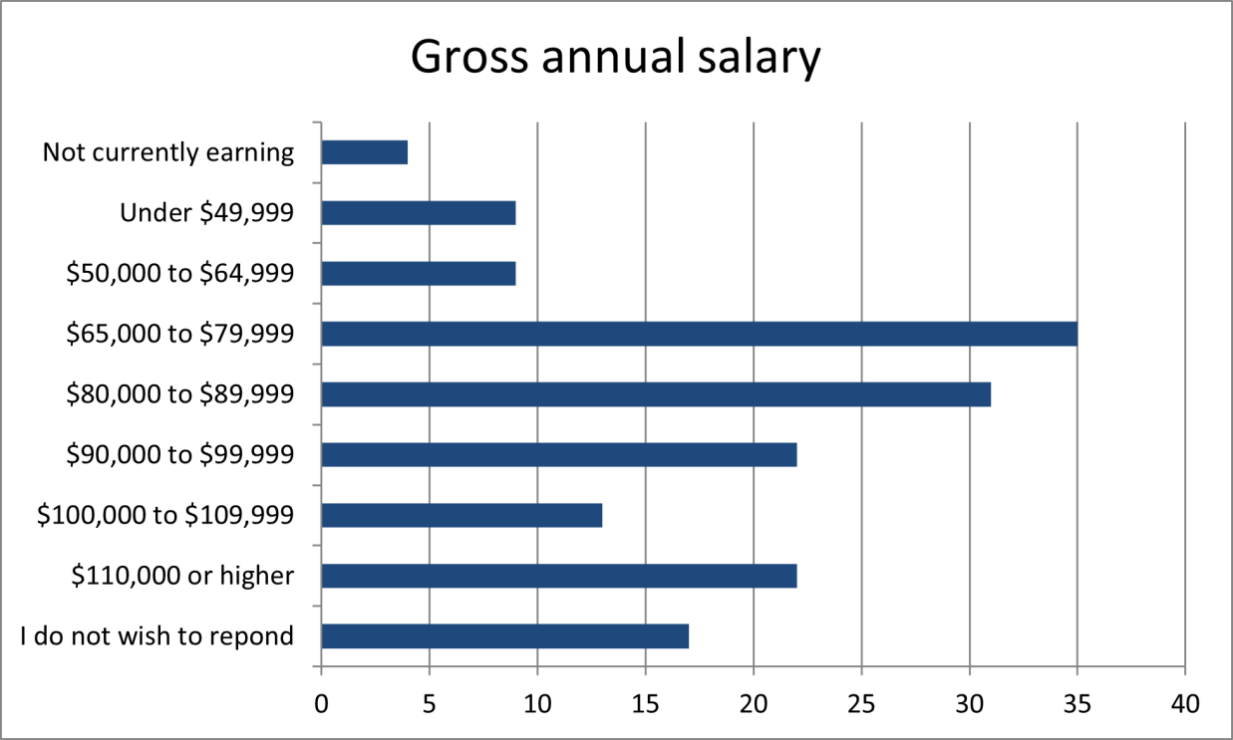
Number of respondents for each salary range

The majority of responses to the question concerning gross annual salary were clustered between $65,000 and $99,999, of which the highest percentage was 22% (N=35) between $65,000 and $79,999. Figure 3 shows the complete range of responses to this question.

### CHLA/ABSC future directions (questions 24-27)

“[I]t is not about just doing one thing and checking a box, it is about mindfully ensuring as an organization we are working with a focus on EDI on all things” (Anonymous survey response, 2023).

The vast majority of respondents wanted CHLA/ABSC to provide more CE sessions on EDI (86%, N=140), with 22 respondents saying that EDI should not be the focus (14%) of the association. Responses are summarized in Table 2.

**Table 2:** Respondents’ recommendations for the CHLA/ABSC’s role with regards to EDI*

	Percentage of responses	Number of respondents
Grants/Scholarships	62%	100
Continuing Education (CE)	86%	140
Resource Sharing	60%	97
Conference Offerings (e.g., keynote speakers, special events)	85%	137
Other (please specify)	9%	15
**Answered**		162
**Skipped**		5

* Each respondent could select more than one option

22 respondents indicated they would not like CHLA/ABSC to offer more EDI-focused CE. Explanations provided suggested that they felt that CE sessions are not enough, and that more systemic changes are needed such as changing hiring practices, diversifying our profession, and providing opportunities to underrepresented groups to be mentored and for networking. This is an example of a response:

I think there should be some thoughtfulness towards EDI in all facets of CHLA, for example pricing for the conference for equity-deserving groups or maybe take that into perspective with other CE pricing or membership opportunities. Personally, I would seek out EDI CE from outside CHLA, unless there was a specific link to health sciences librarianship and information practices.

When asked to specify EDI related topics for possible future CE sessions, the following were mentioned: workplace-related training to provide better services such as training for front-end staff, anti-racism training, de-centering whiteness in libraries, micro-aggression, ableism, accessibility, and inequity in the profession. Other topics mentioned included allyship (N=13); Indigenous knowledge, resources or topics (N=10); improving collections such as decolonization of collections and improving subject headings (N=10); instruction with an EDI lens (N=6); improving the website and/or conference for members with non-visible barriers/disabilities (N=4); speakers to address antisemitism (N=2), and women in libraries (N=1).

### Personal experiences and reflections (questions 28-31)

Question 28 asked participants to reflect back on their past CHLA/ABSC experiences with conferences and activities.

Welcome and inclusion at CHLA/ABSC events. 78% of respondents agreed that they feel welcome and included at CHLA/ABSC events (Strongly agree N=54, Agree N=66). However, 21% of respondents (N=32) said they neither agreed nor disagreed with the statement, while one respondent disagreed.

Treatment with respect at CHLA/ABSC events. 84% of respondents agreed that they are treated with respect at CHLA/ABSC events (Strongly agree N=66, Agree N=63). However, 15% (N=23) said they neither agreed nor disagreed with the statement, while one respondent disagreed. Participation level within CHLA/ABSC. 82% of respondents agreed that there are opportunities within CHLA/ABSC to participate at levels they feel comfortable with (Strongly agree N=49, Agree N=77). However, 16% (N=24) said they neither agreed nor disagreed with the statement, while three respondents disagreed.

Comfort sharing personal experiences within CHLA/ABSC. 65% of respondents agreed that they feel comfortable sharing their personal perspectives and experiences within CHLA/ABSC (Strongly agree N=40, Agree N=59). However, 29% (N=44) said they neither agreed or disagreed with the statement, while 6% (N=9) disagreed, and one respondent strongly disagreed.

Question 29 asked about what CHLA/ABSC can do to improve, based on their reflections on recent CHLA/ABSC experiences. The most common suggestions were about providing CE or workshops or inviting guest speakers; making the annual conference and other events more accessible to members; and making opportunities for everyone to participate in conferences, on the Board, and in the profession in general.

In the same vein, question 30 asked what CHLA/ABSC should continue doing. Respondents encouraged CHLA/ABSC to continue engaging members in the EDI conversation, providing networking and professional development opportunities, ensuring the conference is welcoming and accessible, and having diverse guest speakers.

Finally, question 31 asked respondents if there was anything else they would like to share. In this section, there were a number of praises for the survey as a first step in the right direction to gather feedback.

## Discussion

The demographic data from the survey illustrates a homogeneous group of professionals who were primarily CHLA/ABSC members or members of its Chapters across Canada. Most of the respondents were librarians with Master’s or PhD degrees working at a post-secondary institution or hospital. Many have access to professional development funds, and most had not participated in formal mentorship programs. The majority of survey respondents did not identify as 2SLGBTQIA+, and did not consider themselves as belonging to visible minority, Indigenous, or disabled groups.

While the majority of respondents felt welcomed and respected within CHLA/ABSC events, there is room for improvement. Gathering further feedback from members would provide direction on how CHLA/ABSC can evolve and create a welcoming and respectful environment for health information professionals and the communities they serve. CHLA/ABSC could also explore ways for diverse members to participate in leadership positions within the association.

Members recommended that CHLA/ABSC should continue to listen and improve its EDI work by engaging and collaborating with members to deliver current and relevant networking and CE opportunities. However, CHLA/ABSC must balance its CE offerings with training focusing on professional skills and competencies, in order to ensure that its activities are useful to its members.

Given these results, the EDITF recommends that CHLA/ABSC undertake the following action items to advance EDI within the association:

- Retire the EDITF and create a standing committee to assist the Board in advancing EDI objectives within the association.
- Incorporate EDI into CHLA/ABSC’s strategic and communications plans and include assessment and evaluation measures of EDI activities and initiatives to ascertain if goals and outcomes are achieved.
- Advance EDI and social justice initiatives by developing guidelines for collections, instruction, and reference services.
- Collaborate with other organizations on diversity and inclusion issues and causes.
- Provide guidance to the Conference Planning and CE committees to incorporate EDI into their work.
- Encourage EDI training to the Board of Directors so that EDI issues can guide their work.
- Create conversational opportunities with CHLA/ABSC members about EDI through various mediums such as surveys, focus groups, and other social media platforms.
