# Supplementary material for: Understanding equity and diversity needs among health library professionals in Canada: a survey
Source: J Can Health Libr Assoc. 2024 Apr 1;45(1):44–51. doi: 10.29173/jchla29700 (PMC11081120; doi:10.29173/jchla29700)
Supplement: Supplementary file 1 — Online Supplement Appendix [file JCHLA-45-044-s001.pdf]

Appendix 1: 2023 CHLA/ABSC Equity, Diversity and Inclusion (EDI) Survey (English version)

Q1: Are you currently a member of CHLA/ABSC?
Answer Choices: Yes/No

Q2: Are you currently a member of a CHLA/ABSC Chapter?
Answer Choices: Yes/No

Q3: Which one?
Answer Choices:
- FMD3S
- Golden Horseshoe Health Libraries Association
- Health Libraries Association of British Columbia
- Manitoba Association of Health Information Providers
- Maritimes Health Libraries Association / Association des bibliothèques de la santé des Maritimes
- Newfoundland and Labrador Health Libraries Association
- Northern Alberta Health Libraries Association
- Ottawa Valley Health Libraries Association / Association des bibliothèques de la santé de la Vallée d'Outaouais
- Saskatchewan Health Libraries Association
- Southern Alberta Health Libraries Association
- Toronto Health Libraries Association
- Wellington-Waterloo-Dufferin Health Library Network

Q4: Are you currently employed in a library position?
Answer Choices: Yes/No

Q5: What is the nature of your employment? Select all that apply
Answer Choices:
- Part-time (one position)
- Part-time (multiple positions)
- Full-time permanent
- Full-time contract
- Self-employed
- Other (please specify)

Q6: What is your primary position?
Answer Choices:
- Librarian
- Library Assistant/Library Technician
- Information Specialist
- Administrator/Management
- Other (please specify)

Q7: What type of library/organization do you work for? Select all that apply.
Answer Choices

- Post-secondary institution (e.g., university, college)
- Consortia
- Hospital
- Publisher
- Special
- Public
- Other (please specify)

Q8: Is your job specific to the health sciences?
Answer Choices: Yes/No

Q9: Are you currently part of a union?
Answer Choices: Yes/No

Q10: Are you currently AHIP (Academy of Health Information Professionals) certified?
Answer Choices: Yes/No

Q11: Do you plan to pursue AHIP certification?
Answer Choices: Yes/No/Undecided/Unsure

Q12: Does your employer provide funds for professional development?
Answer Choices: Yes/No

Q13: How do you access those funds?
Answer Choices

- Via a personal PD fund that I can use at my discretion
- Via a group fund that I must obtain approval for

Q14: Have you participated in any formal mentorship programs that you would recommend to a colleague?
Answer Choices

- No/Yes (please specify)

Q15: Do you consider yourself as belonging to a visible minority group? (ViMLoC specifies that visible minority populations consist mainly of the following groups: Chinese, South Asian, Black, Filipino, Arab, West Asian, Southeast Asian, Latin American, Japanese and Korean.)
Answer Choices: Yes/No/I do not wish to respond

Q16: Do you consider yourself to be of Indigenous ancestry? (Definition: “Indigenous ancestry” refers to whether a person has ancestry associated with First Nations, Métis, and/or Inuit)
Answer Choices: Yes/No/I do not wish to respond

Q17: Do you consider yourself to be a person with a disability? Select all that apply.(Note: the social model of disability recognizes that disability is not created by any particular medical or physical condition, but rather by societal barriers.)
Answer Choices:

- Sensory (e.g., Seeing, Hearing)
- Physical (e.g., Mobility, Flexibility, Dexterity, Chronic Pain)
- Cognitive (e.g., Learning, Developmental, Memory)
- Mental Health-Related (e.g., Depression, Anxiety, ADHD)
- Other/Unknown
- I do not consider myself to be a person with a disability
- I do not wish to respond

Q18: Do you identify as 2SLBGTQIA+? (Definition: Two-Spirit, Lesbian, Gay, Bisexual, Transgender, Queer or Questioning, Intersex, Asexual, and additional sexual orientations and gender identities)
Answer Choices: Yes/No/I do not wish to respond

Q19: What is your age group?
Answer Choices:

- Under 25
- 25-34
- 35-44
- 45-54
- 55-64
- Over 65

Q20: Where do you work?
Answer Choices:

- Alberta
- British Columbia
- Manitoba
- New Brunswick
- Newfoundland and Labrador
- Northwest Territories
- Nova Scotia
- Nunavut
- Ontario
- Prince Edward Island
- Quebec
- Saskatchewan
- Yukon
- Other/I work remotely (please specify)

Q21: How many years of work experience in health-related libraries or as an information professional in the health industry do you have?
Answer Choices:

- Less than 1 year
- 1-5 years
- 6-10 years
- 11-15 years
- More than 15 years

Q22: What is the highest level of formal education in library/information science you have or are currently completing?
Answer Choices:

- Certificate program
- College/University diploma
- Master's Degree
- Doctorate/PhD
- Other (please specify)

Q23: As of January 2023, what is your gross annual salary (before taxes and other deductions)? [Amounts are in Canadian dollars. International respondents please adjust accordingly.]
Answer Choices:

- Not currently earning
- Under $49,999
- $50,000 to $64,999
- $65,000 to $79,999
- $80,000 to $89,999
- $90,000 to $99,999
- $100,000 to $109,999
- $110,000 or higher
- I do not wish to respond

Q24: What do you think CHLA/ABSC's role should be in regards to EDI?
Answer Choices:

- Grants/Scholarships
- Continuing Education (CE)
- Resource Sharing
- Conference offerings (e.g., keynote speakers, special events)
- Other (please specify)

Q25: Would you like CHLA/ABSC to offer more CE sessions related to EDI?
Answer Choices: Yes/No

Q26: Why not?
Open ended question

Q27: Please specify which EDI related topics would most interest you in a CHLA/ABSC CE session?
Open ended question

Q28: Please reflect back on your most recent CHLA/ABSC experiences and respond to the following statements

For each statement respondents could select one of the five options from a scale of: Strongly agree; Agree; Neither agree nor disagree; Disagree; Strongly Disagree)

- I have felt welcome and included at CHLA/ABSC events
- I feel I am treated with respect at CHLA/ABSC events
- There are opportunities within CHLA/ABSC for me to participate at a level I feel comfortable with
- I feel comfortable sharing my personal perspective and experiences within CHLA/ABSC

Q29: Based on your answers above, what do you think CHLA/ABSC could do to improve?
Open ended question

Q30: Based on your answers above, what do you think CHLA/ABSC should continue doing?
Open ended question

Q31: Is there anything else you would like to share?
Open ended question

2023 CHLA/ABSC Equity, Diversity and Inclusion (EDI) Survey (French version)

Q1: Êtes-vous présentement membre de l'ABSC / CHLA ?
Choix de réponse : Oui/Non

Q2: Êtes-vous présentement membre d'une section de l'ABSC / CHLA ?
Choix de réponse : Oui/Non

Q3: Laquelle ?
Choix de réponse :

- FMD3S
- Golden Horseshoe Health Libraries Association
- Health Libraries Association of British Columbia

- Manitoba Association of Health Information Providers
- Maritimes Health Libraries Association / Association des bibliothèques de la santé des Maritimes
- Newfoundland and Labrador Health Libraries Association
- Northern Alberta Health Libraries Association
- Ottawa Valley Health Libraries Association / Association des bibliothèques de la santé de la Vallée d'Outaouais
- Saskatchewan Health Libraries Association
- Southern Alberta Health Libraries Association
- Toronto Health Libraries Association
- Wellington-Waterloo-Dufferin Health Library Network

Q4: Occupez-vous actuellement un poste dans une bibliothèque ?
Choix de réponse : Oui/Non

Q5: Quelle est la nature de votre emploi ? Sélectionnez toutes les réponses qui s'appliquent
Choix de réponse :

- Temps partiel (un poste)
- Temps partiel (plusieurs postes)
- Temps plein permanent
- Temps plein temporaire
- Travailleur(-euse) autonome
- Autre (veuillez préciser)

Q6: Quelle est votre fonction principale ?
Choix de réponse :

- Bibliothécaire
- Assistant(-e) de bibliothèque / Technicien(-ne) en documentation
- Spécialiste de l'information
- Administration / Gestion
- Autre (veuillez préciser)

Q7: Pour quel type de bibliothèque ou d'organisation travaillez-vous ? Sélectionnez toutes les réponses qui s'appliquent.
Choix de réponse :

- Institution post-secondaire (p. ex. université, collège)
- Consortium
- Hôpital
- Éditeur
- Spéciale
- Publique
- Autre (veuillez spécifier)

Q8: Votre emploi est-il spécifique aux sciences de la santé ?

Choix de réponse : Oui/Non

Q9: Faites-vous actuellement partie d'un syndicat ?
Choix de réponse : Oui/Non

Q10: Détenez-vous une certification AHIP (Academy of Health Information Professionals) ?
Choix de réponse : Oui/Non

Q11: Envisagez-vous d'obtenir la certification AHIP ?
Choix de réponse : Oui/Non/Indécis(-e) / Incertain(-e)

Q12: Votre employeur fournit-il des fonds pour le développement professionnel ?
Choix de réponse : Oui/Non

Q13: Comment accédez-vous à ces fonds ?
Choix de réponse
- Via un fonds personnel que je peux utiliser à ma discrétion
- Via un fonds de groupe pour lequel je dois obtenir une approbation

Q14: Avez-vous participé à des programmes de mentorat formel que vous recommanderiez à un(-e) collègue ?
Choix de réponse
- Non/Oui (veuillez spécifier)

Q15: Vous considérez-vous comme appartenant à une minorité visible ? (ViMLoC spécifie que les populations des minorité visibles se composent principalement des groupes suivants : Chinois, Asiatiques du Sud, Noirs, Philippins, Arabes, Asiatiques de l'Ouest, Asiatiques du Sud-Est, Latino-Américains, Japonais et Coréens.)
Choix de réponse : Oui/Non/Je ne veux pas répondre

Q16: Vous considérez-vous comme étant d'ascendance autochtone ? (Définition : « ascendance autochtone » indique si la personne a une ascendance liée aux Premières Nations, aux Métis et/ou aux Inuits)
Choix de réponse : Oui/Non/Je ne veux pas répondre

Q17: Vous considérez-vous comme étant une personne ayant une incapacité ? Sélectionnez toutes les réponses pertinentes.(Note : le modèle social de l'incapacité reconnaît que l'incapacité n'est pas créée par une condition médicale ou physique particulière, mais plutôt par des obstacles imposés par la société)
Choix de réponse :
- Sensorielle (p. ex. Vue, Ouïe)
- Physique (p. ex. Mobilité, Flexibilité, Dextérité, Douleur)
- Cognitive (p. ex. Apprentissage, Développement, Mémoire)

- Santé mentale (p. ex. Dépression, Anxiété, Problèmes d'attention)
- Autre / Inconnu
- Je ne me considère pas comme une personne ayant une incapacité
- Je ne veux pas répondre

Q18: Vous identifiez-vous comme 2SLBGTQIA+ ?(Définition : Deux-esprits, Lesbienne, Gaie, Bisexuelle, Transgenre, Queer, Intersexe, Asexuelle, et autres orientations sexuelles et identités de genre)
Choix de réponse : Oui/Non/Je ne veux pas répondre

Q19: Quel est votre groupe d'âge ?
Choix de réponse :
- Moins de 25 ans
- 25 à 34 ans
- 35 à 44 ans
- 45 à 54 ans
- 55 à 64 ans
- 65 ans et +

Q20: Où travaillez-vous ?
Choix de réponse :
- Alberta
- Colombie britannique
- Île-du-Prince-Édouard
- Manitoba
- Nouveau-Brunswick
- Nouvelle-Écosse
- Nunavut
- Ontario
- Québec
- Saskatchewan
- Terre-Neuve-et-Labrador
- Territoires du Nord-Ouest
- Yukon
- Autre / Je travaille à distance (veuillez préciser)

Q21: Combien d'années d'expérience professionnelle dans des bibliothèques liées à la santé ou en tant que professionnel(-le) de l'information dans le secteur de la santé avez-vous ?
Choix de réponse :
- 1 an ou moins
- 1 à 5 ans
- 6 à 10 ans
- 11 à 15 ans
- Plus de 15 ans

Q22: Quel est le plus haut niveau d'éducation formelle en bibliothéconomie / sciences de l'information que vous avez ou que vous êtes en train de compléter ?
Choix de réponse :

- Certificat
- Diplôme collégial ou universitaire
- Maîtrise
- Doctorat / PhD
- Autre (veuillez préciser)

Q23: En date de janvier 2023, quel est votre salaire annuel brut (avant impôts et autres déductions) ? [Les montants sont en dollars canadiens. Les répondant(-e)s internationaux(-ales) sont prié(-e)s d'ajuster en conséquence.]
Choix de réponse :

- Aucun salaire
- Moins de 49 999 $
- 50 000 $ à 64 999 $
- 65 000 $ à 79 999 $
- 80 000 $ à 89 999 $
- 90 000 $ à 99 999 $
- 100 000 $ à 109 999 $
- 110 000 $ et plus
- Je ne veux pas répondre

Q24: Selon vous, quel devrait être le rôle de l'ABSC / CHLA en ce qui concerne l'EDI ?
Choix de réponse :

- Subventions / Bourses d'études
- Formation continue (FC)
- Partage de ressources
- Contenu de conférence (p. ex. conférencier(-ère)s, événements spéciaux)
- Autre (veuillez préciser)

Q25: Aimeriez-vous que l'ABSC / CHLA offre plus de séances de formation continue sur l'EDI ?
Choix de réponse : Oui/Non

Q26: Pourquoi pas ?
Question ouverte

Q27: Veuillez préciser quels sujets reliés à l'EDI vous intéresseraient le plus dans une séance de formation continue de l'ABSC / CHLA
Question ouverte

Q28: Réfléchissez à vos expériences les plus récentes avec l'ABSC / CHLA et évaluez les affirmations suivantes

Pour chaque énoncé, les répondants pouvaient sélectionner l'une des cinq options sur une échelle de : Fortement d'accord ; D’accord; Ni d'accord ni en désaccord; En désaccord; Fortement en désaccord)

- Je me suis senti(-e) le(la) bienvenu(-e) et inclus(-e) lors d'événements de l'ABSC / CHLA
- Je sens que je suis traité(-e) avec respect lors des événements de l'ABSC / CHLA
- Il y a des opportunités pour moi au sein de l'ABSC / CHLA de participer à un niveau avec lequel je me sens à l'aise
- Je me sens à l'aise de partager mon point de vue et mes expériences personnelles au sein de l'ABSC / CHLA

Q29: Basé sur vos réponses ci-dessus, que pensez-vous que l'ABSC / CHLA pourrait faire pour s'améliorer ?
Question ouverte

Q30: Basé sur vos réponses ci-dessus, que pensez-vous que l'ABSC / CHLA devrait continuer de faire ?
Question ouverte

Q31: Y a t-il autre chose que vous aimeriez partager ?
Question ouverte
