# Supplementary material for: Understanding equity and diversity needs among health library professionals in Canada: a survey
Source: J Can Health Libr Assoc. 2024 Apr 1;45(1):44–51. doi: 10.29173/jchla29700 (PMC11081120; doi:10.29173/jchla29700)
Supplement: Supplementary file 2 — Online Supplement Appendix [file JCHLA-45-044-s002.pdf]

Summary of CHLA/ABSC Survey Results

Question 1: Are you currently a member of CHLA/ABSC?

The total respondents for this question were one hundred and sixty-six. Most of the respondents (N=138, 83.13%) are members of CHLA/ABSC. The rest were not (N=28, 16.87%).

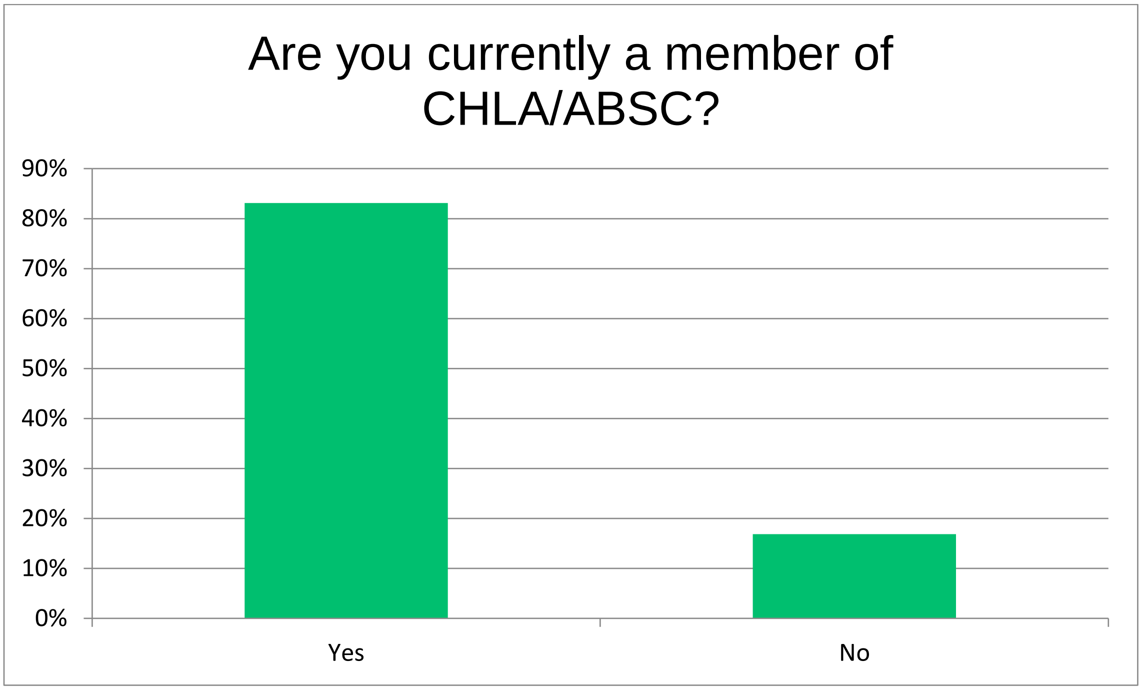

Answer Choices	Percentages	Number
Yes	83.13%	138
No	16.87%	28
	Answered	166
	Skipped	1

Question 2: Are you currently a member of a CHLA/ABSC Chapter?

Many of the respondents are also currently members of CHLA/ABSC Chapters (N=104, 62.65%). The rest were not (N=62, 37.35%). It was not evident from the questions why members have not joined their local chapter.

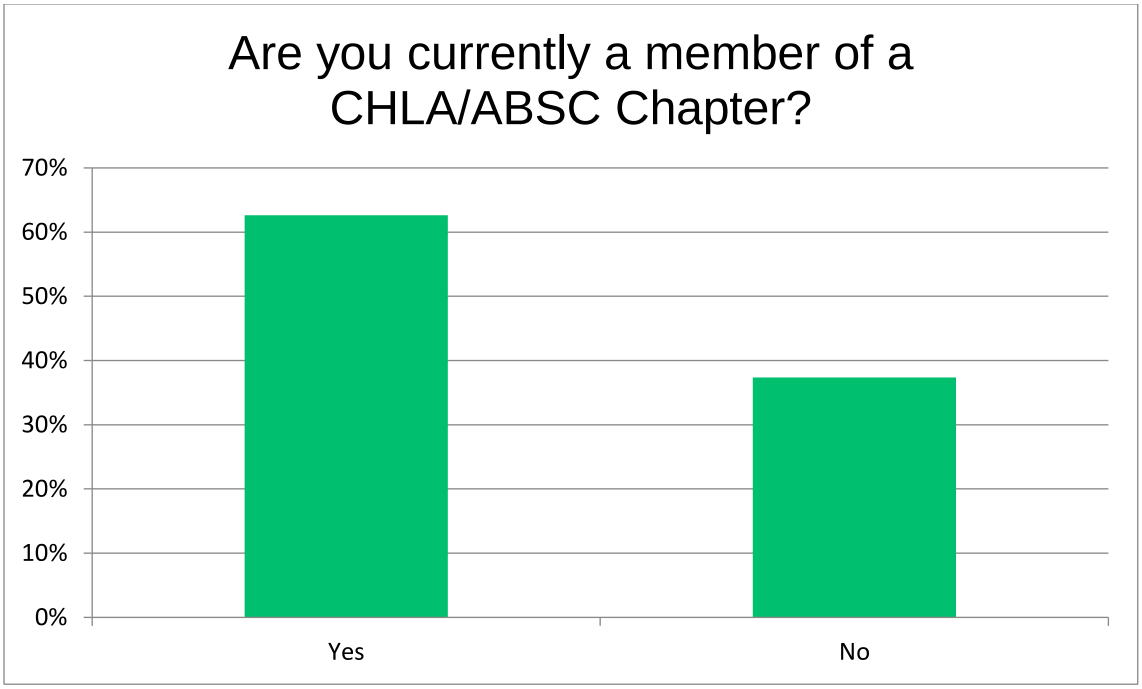

Answer Choices	Percentages	Number
Yes	62.65%	104
No	37.35%	62
	Answered	166
	Skipped	1

Skip to question 4 if "No" is selected.

Question 3: Which one?

All of the members that answered they were part of a chapter in question two, identified which chapter they belong to in this question.

The most popular chapter was Health Libraries Association of British Columbia (N=16, 15.38%); followed by Toronto Health Libraries Association and Maritimes Health Libraries Association (N=15, 14.42%); Ottawa Valley Health Libraries Association (N=13, 12.5%); FMD3S (N=12, 11.54%); Saskatchewan Health Libraries Association (N=11, 10.58%); Northern Alberta Health Libraries Association and Southern Alberta Health Libraries Association (N=7, 6.73%); Manitoba Association of Health Information Providers (N=4, 3.85%); Newfoundland and Labrador Health Libraries Association and Wellington Waterloo-Dufferin Health Library Network (N=2, 1.92%); there were no representatives from the Golden Horseshoe Health Libraries Association (N=0).

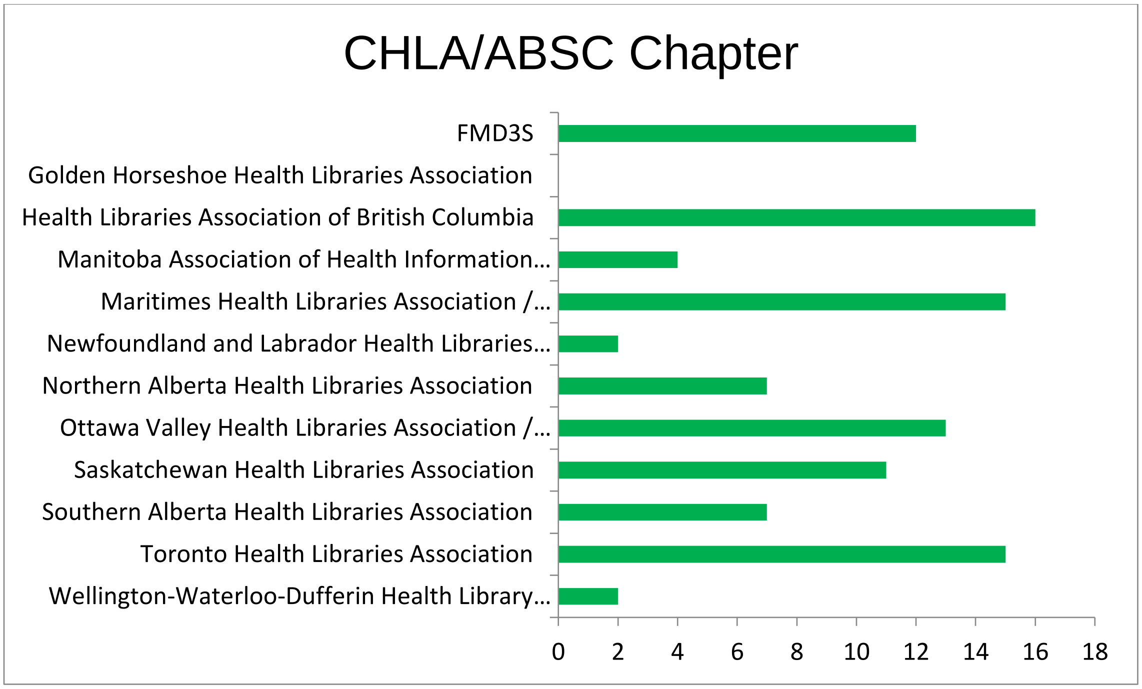

Answer Choices	Percentages	Number
FMD3S	11.54%	12
Golden Horseshoe Health Libraries Association	0%	0
Health Libraries Association of British Columbia	15.38%	16
Manitoba Association of Health Information Providers	3.85%	4
Maritimes Health Libraries Association / Association des bibliothèques de la santé des Maritimes	14.42%	15

Newfoundland and Labrador Health Libraries Association	1.92%	2
Northern Alberta Health Libraries Association	6.73%	7
Ottawa Valley Health Libraries Association / Association des bibliothèques de la santé de la Vallée d'Outaouais	12.50%	13
Saskatchewan Health Libraries Association	10.58%	11
Southern Alberta Health Libraries Association	6.73%	7
Toronto Health Libraries Association	14.42%	15
Wellington-Waterloo-Dufferin Health Library Network	1.92%	2
	Answered	104
	Skipped	63

Question 4: Are you currently employed in a library position?

The majority of respondents work in a library position (N=152, 91.57%).

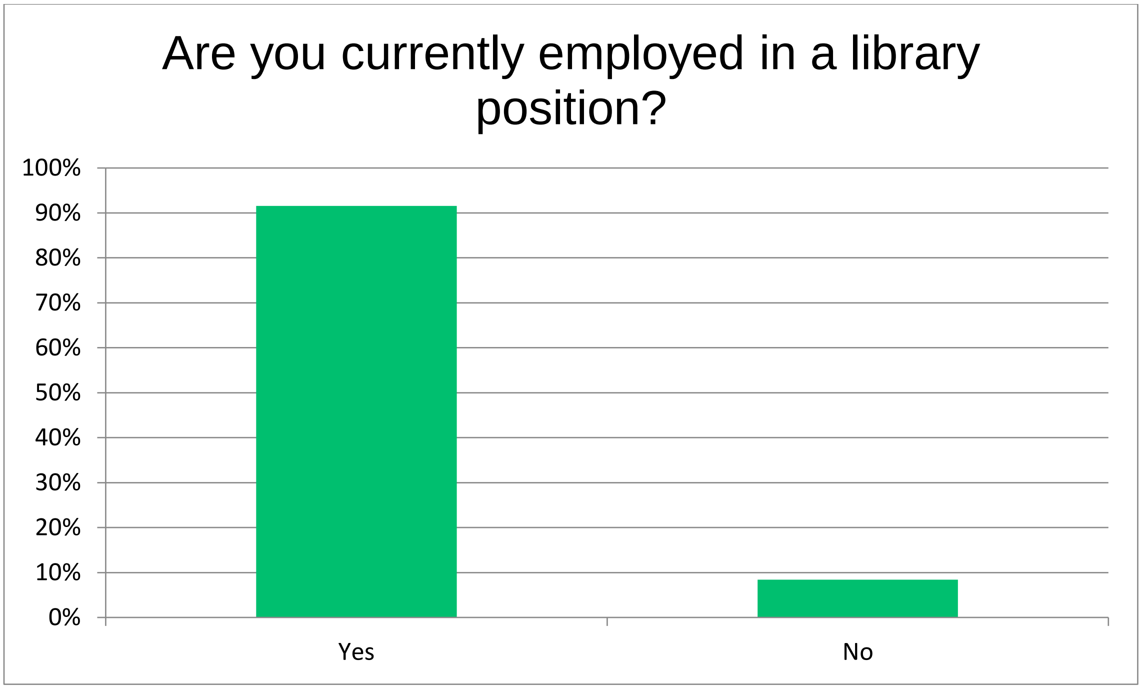

Answer Choices	Percentages	Number
Yes	91.57%	152
No	8.43%	14
	Answered	166
	Skipped	1

Question 5: What is the nature of your employment? Select all that apply

Most of the respondents are full time permanent (N=138, 91.39%); some were part-time in one position (N=7, 4.64%); full-time contract (N=6, 3.97%); one was self-employed (.66%) and another (0.66%) specified part-time contract. None of the respondents were part-time in multiple positions.

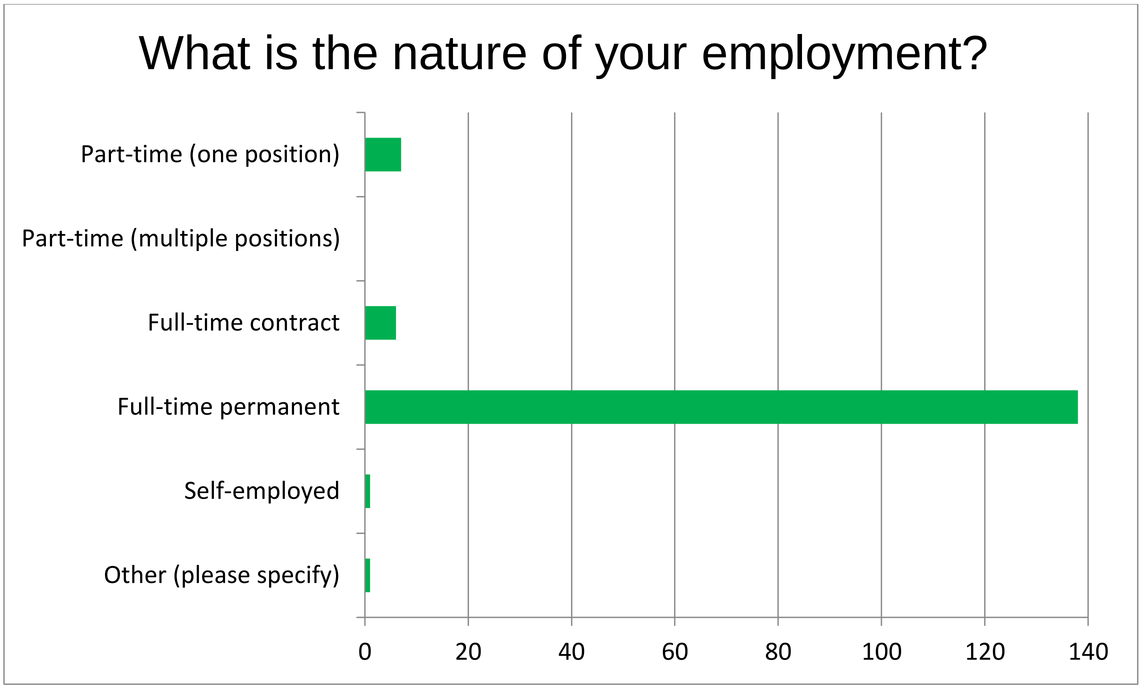

Answer Choices	Percentages	Number
Part-time (one position)	4.64%	7
Part-time (multiple positions)	0%	0
Full-time permanent	91.39%	138
Full-time contract	3.97%	6
Self-employed	0.66%	1
Other (please specify)	0.66%	1
	Answered	151

	Skipped	16

Question 6: What is your primary position?

The majority of the respondents (N=101, 66.89%) are librarians; followed by administrator/manager (N=24, 15.89%); library assistant/technicians (N=12 each, 7.95%), information specialist (N=12 each, 7.95%); and two were other (1 librarian and management and the other program coordinator). One of the "Other" respondents was a program coordinator.

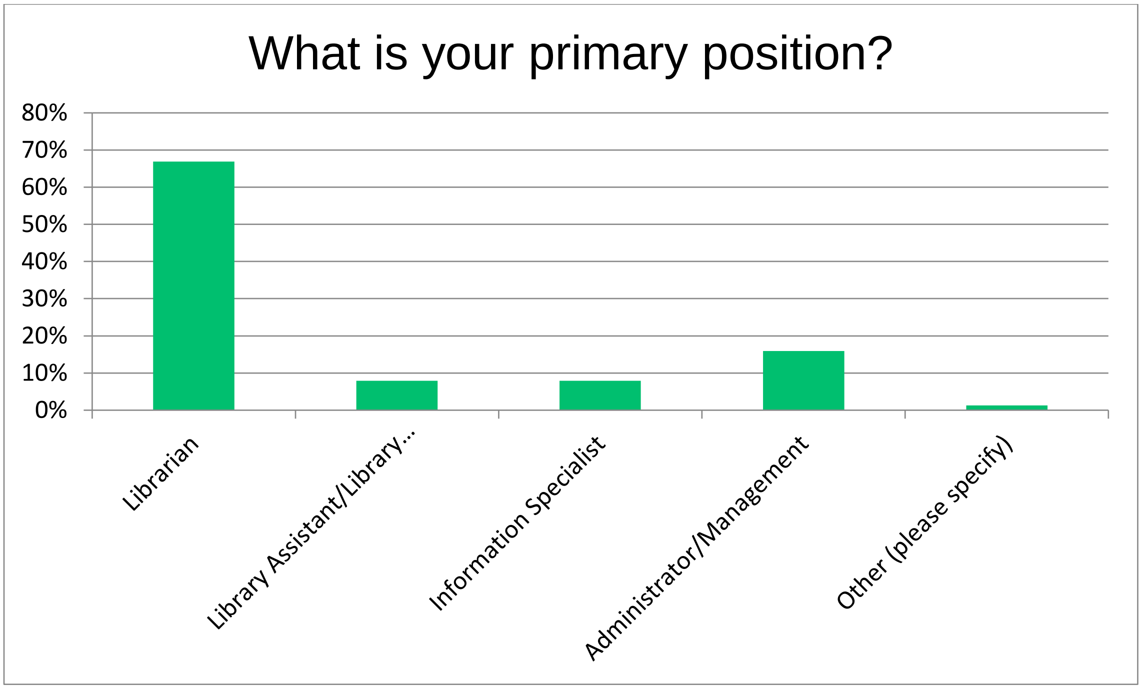

Answer Choices	Percentages	Number
Librarian	66.89%	101
Library Assistant/Library Technician	7.95%	12
Information Specialist	7.95%	12
Administrator/Management	15.89%	24
Other (please specify)	1.32%	2

	Answered	151
	Skipped	16

Question 7: What type of library/organization do you work for? Select all that apply.

The majority of respondents work in a hospital (N=65, 43.05%) and post-secondary institution (N=64, 42.38%). Followed by special libraries (N=20, 13.25%); consortia (N=3, 1.99%); publisher (N=2, 1.32%); and public library (N=1,). Eight (5.30%) respondents said they work in other libraries/organizations as follows: non-for profit, government, health research organization, healthcare outside of hospital, and public health.

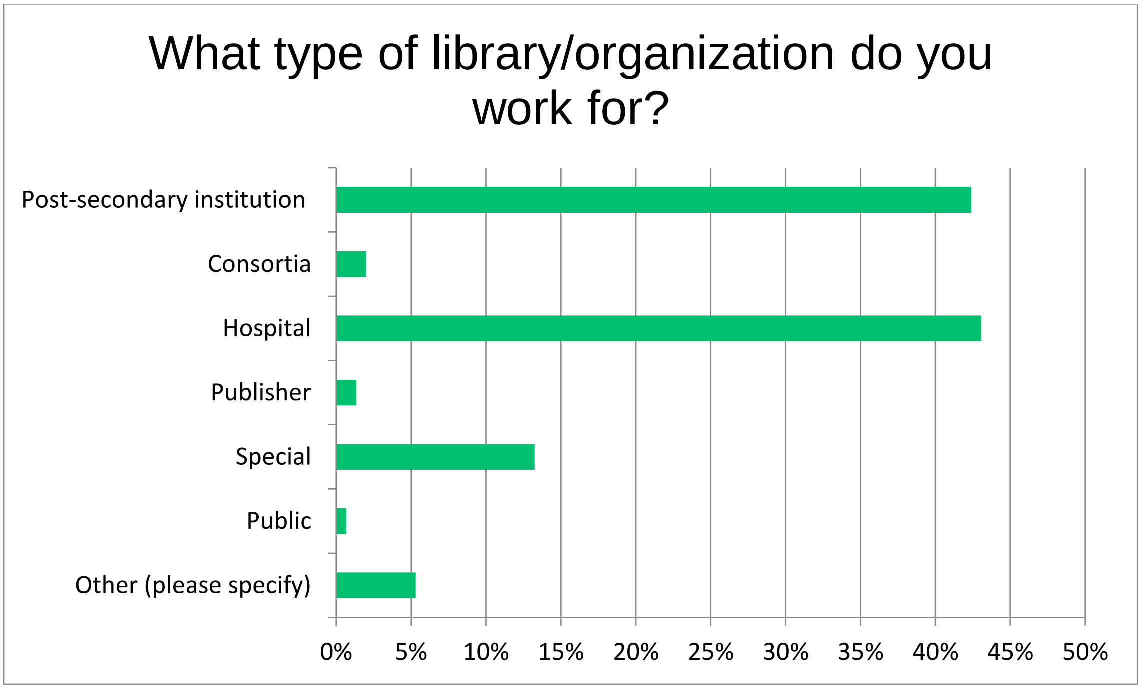

Answer Choices	Percentages	Number
Post-secondary institution	42.38%	64
Consortia	1.99%	3
Hospital	43.05%	65

Publisher	1.32%	2
Special	13.25%	20
Public	0.66%	1
Other (please specify)	5.30%	8
	Answered	151
	Skipped	16

Question 8: Is your job specific to the health sciences?

The majority of respondents work in health sciences (N=134, 89.93%).

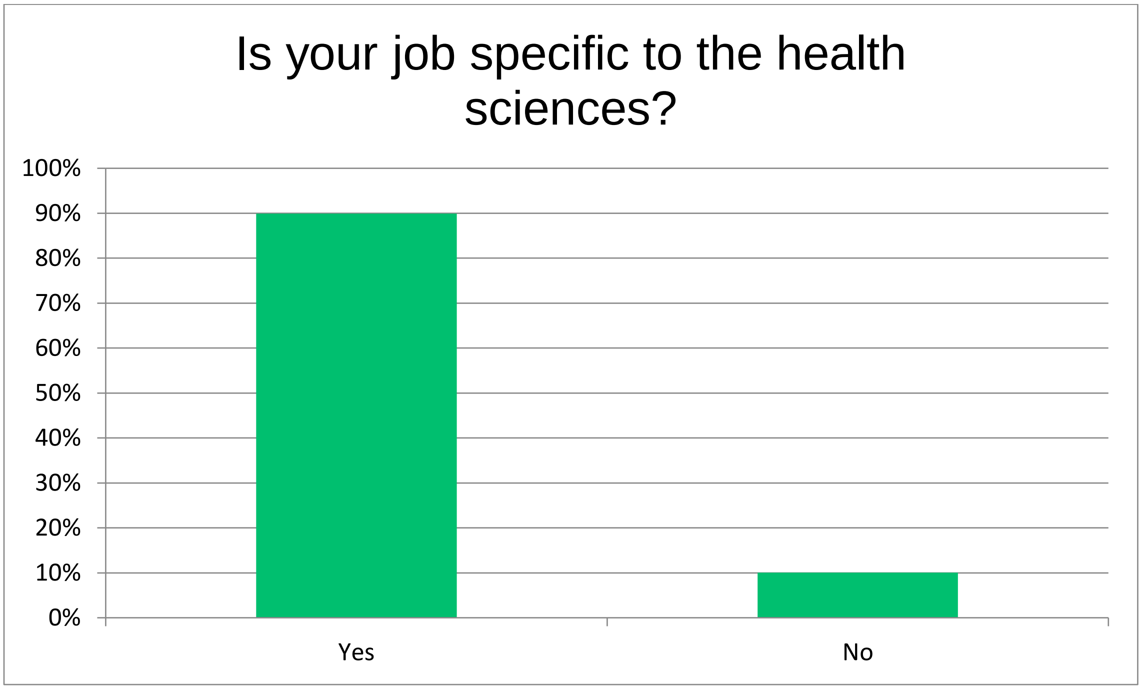

Answer Choices	Percentages	Number
Yes	89.93%	134

No	10.07%	15
	Answered	149
	Skipped	18

Question 9: Are you currently part of a union?

Seventy-nine (52.32%) are unionized and seventy-two are not (47.68%).

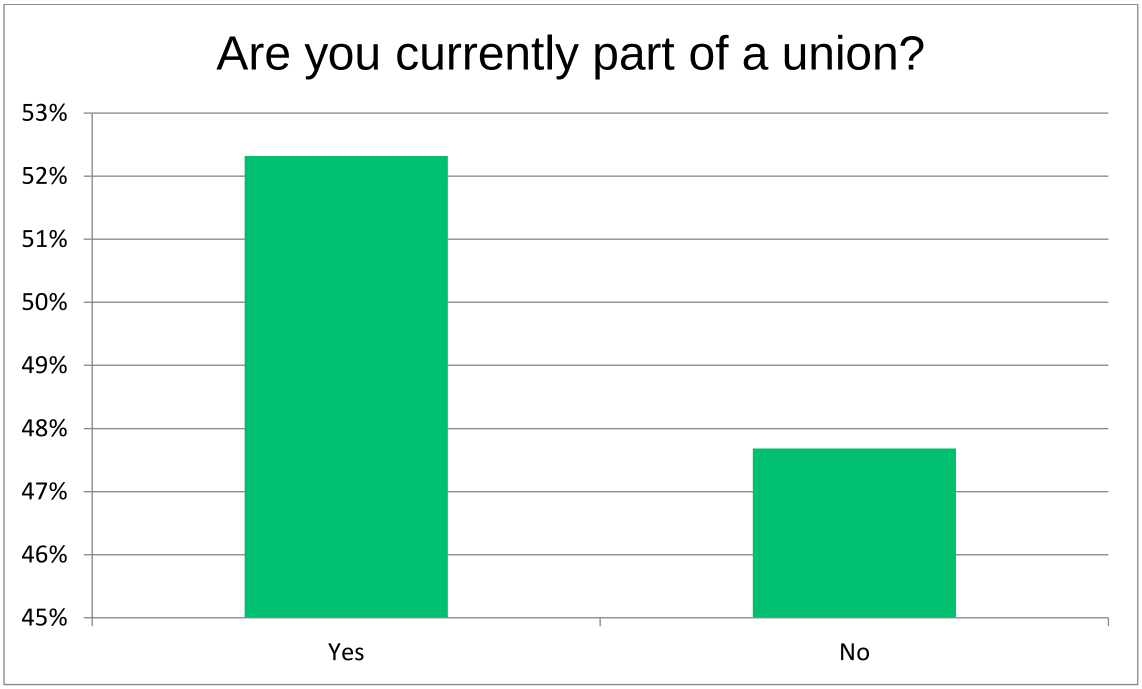

Answer Choices	Percentages	Number
Yes	52.32%	79
No	47.68%	72
	Answered	151
	Skipped	16

Question 10 : Are you currently AHIP (Academy of Health Information Professionals) certified?

One hundred and sixty-five people answered the question. The majority of respondents are not AHIP certified (N=159, 96.36%).

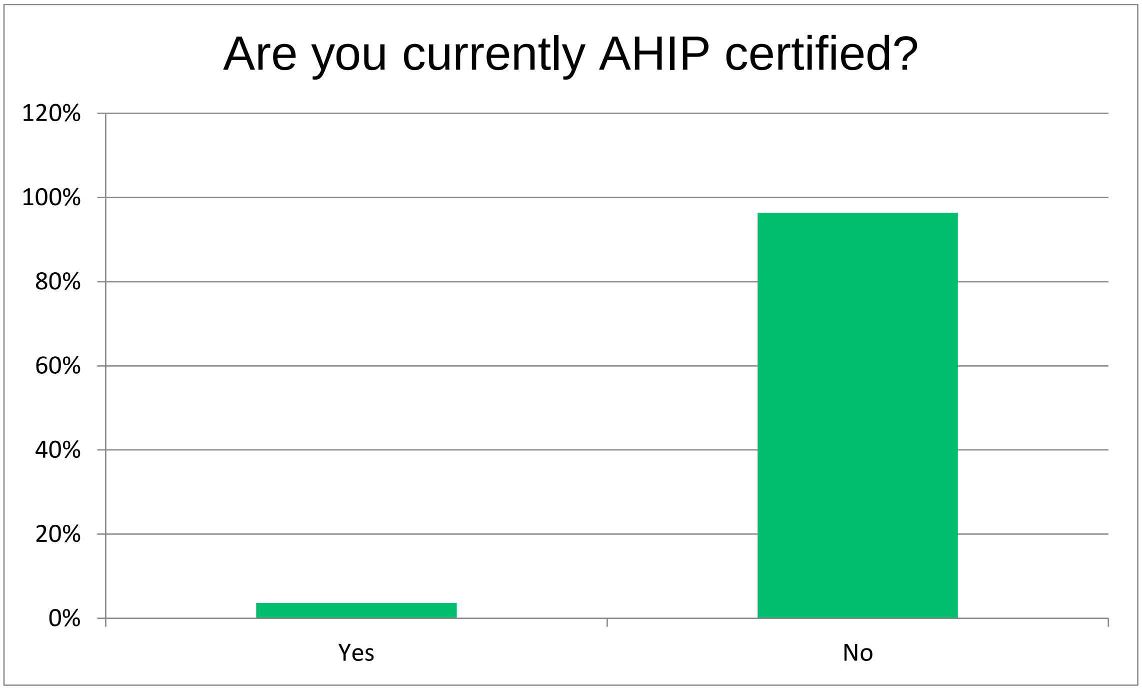

Answer Choices	Percentages	Number
Yes	3.64%	6
No	96.36%	159
	Answered	165
	Skipped	2

Question 11: Do you plan to pursue AHIP certification?

There were hundred and fifty-eight responses to this question. Ninety-one said they do not plan to pursue AHIP (57.59%) and nine said they would (5.70%). Fifty-eight respondents were undecided/unsure (36.71%).

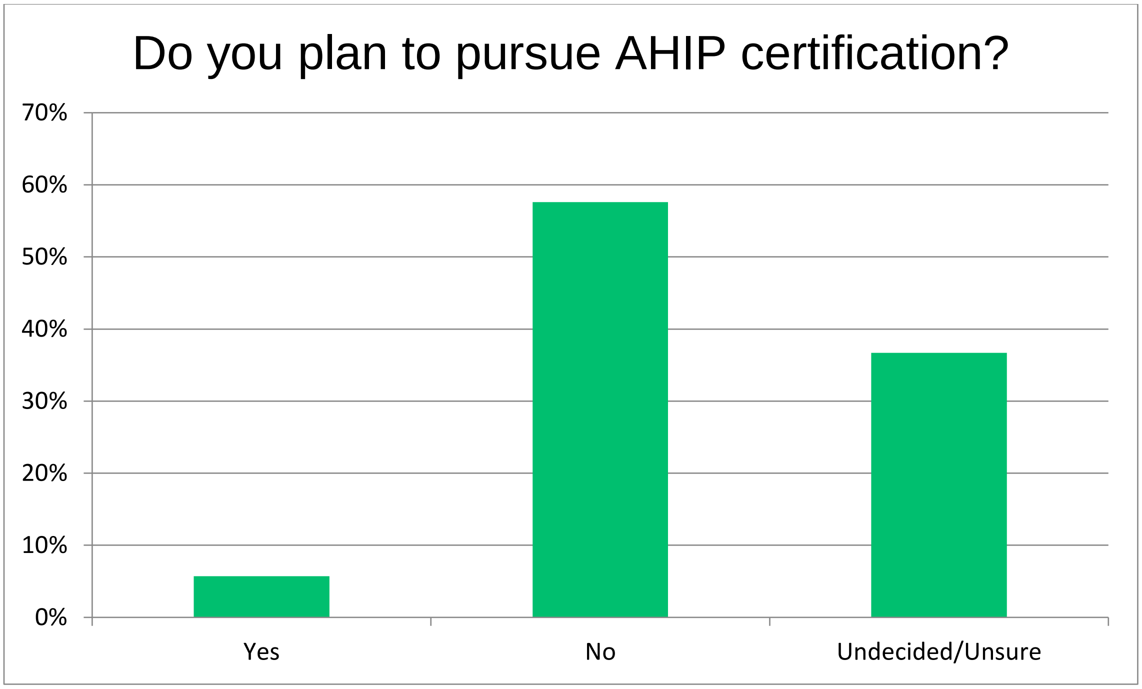

Answer Choices	Percentages	Number
Yes	5.70%	9
No	57.59%	91
Undecided/Unsure	36.71%	58
	Answered	158
	Skipped	9

Question 12: Does your employer provide funds for professional development?

One hundred and sixty-one people responded to this question. Most respondents have funds for professional development (N=135, 83.85%) and twenty-six (N=16.15%) do not.

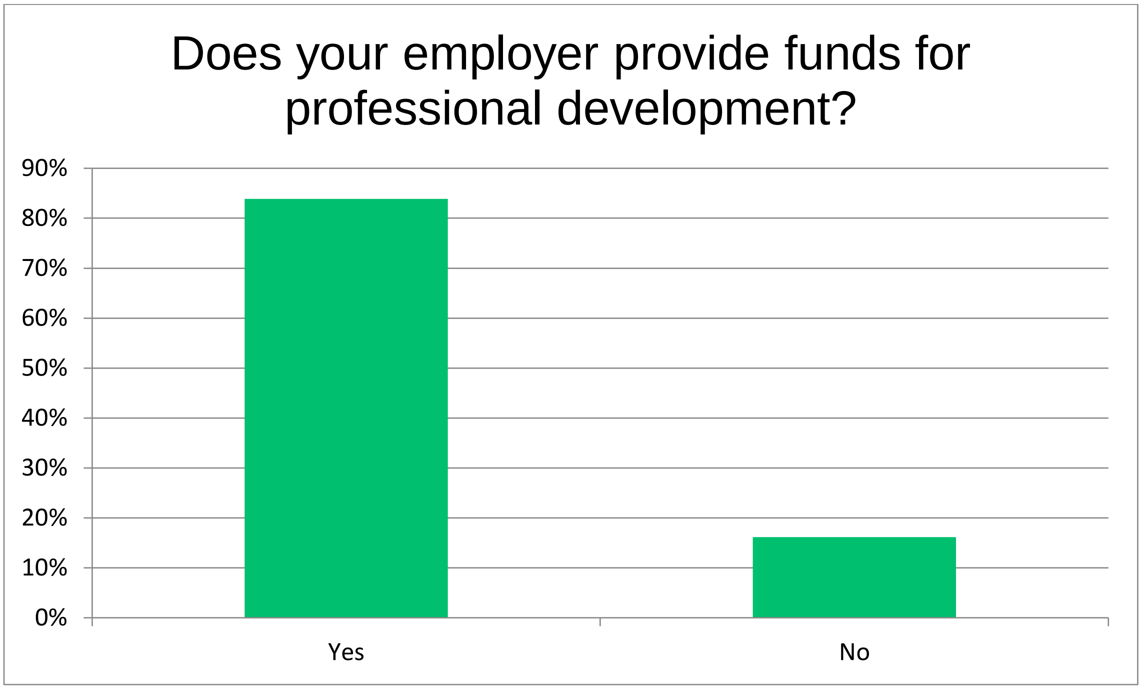

Answer Choices	Percentages	Number
Yes	83.85%	135
No	16.15%	26
	Answered	161
	Skipped	6

Question 13: How do you access those funds?

One hundred and thirty-three respondents answered this question. Most respondents said they have a group fund that they must obtain approval for (N=77, 57.89%), and fifty-six (42.11%) said they have access via a personal fund that they can use at their discretion.

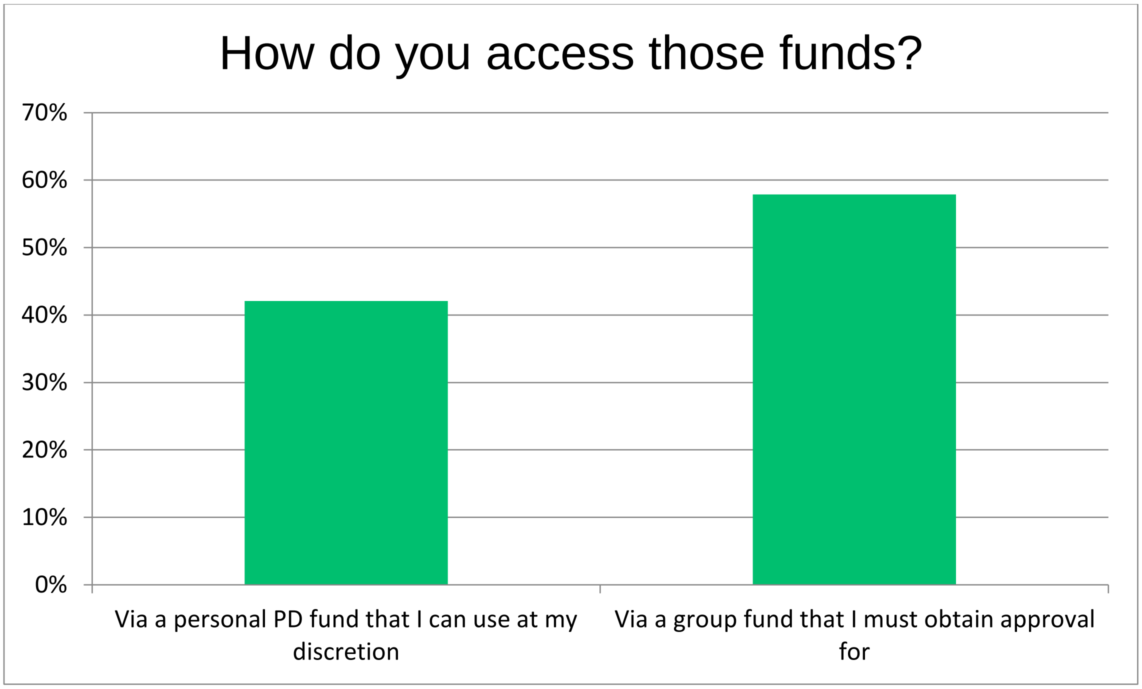

Answer Choices	Percentages	Number
Via a personal PD fund that I can use at my discretion	42.11%	56
Via a group fund that I must obtain approval for	57.89%	77
	Answered	133
	Skipped	34

Question 14: Have you participated in any formal mentorship programs that you would recommend to a colleague?

The majority of respondents (N=138, 86.79%) had not participated in a formal mentorship program that they would recommend to a colleague. However, twenty-one (13.21%) had and provided their recommendations.

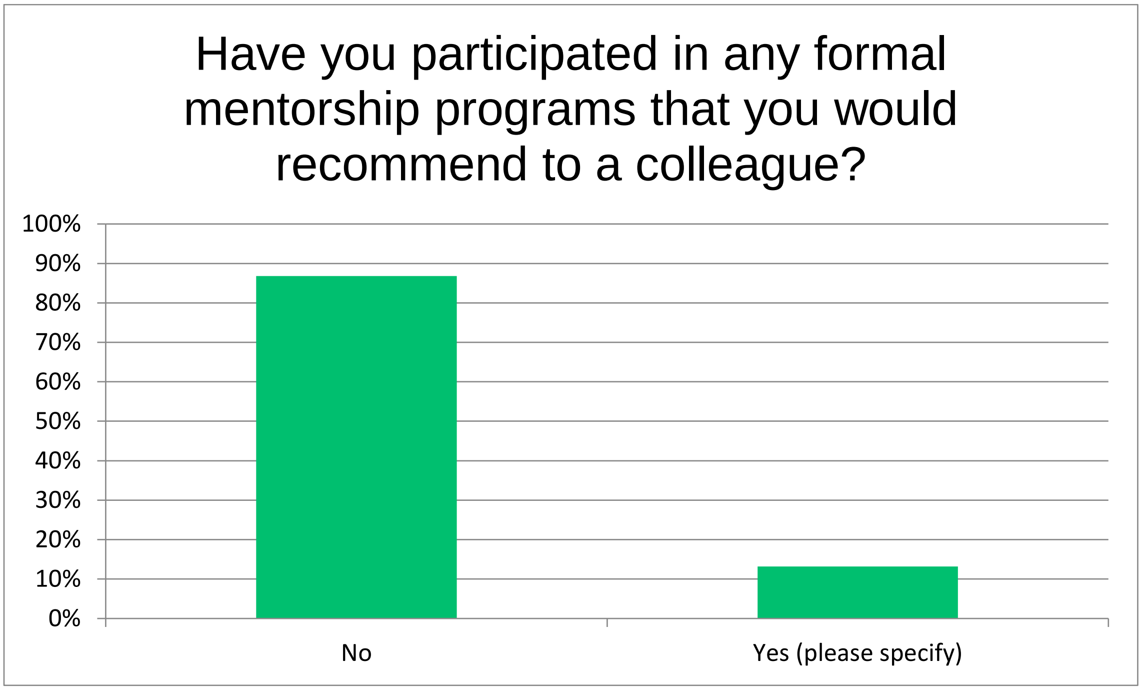

Answer Choices	Percentages	Number
No	86.79%	138
Yes (please specify)	13.21%	21
	Answered	159
	Skipped	8

The following programs were recommended by respondents who had participated in mentorship programs: CAPAL's research mentorship; ACRL Instruction Section mentorship program; ViMLoC; MLA; OLA; ABQLA; CHLA/ABSC Leadership Institute; NELI.

Question 15: Do you consider yourself as belonging to a visible minority group? (ViMLoC specifies that visible minority populations consist mainly of the following groups: Chinese, South Asian, Black, Filipino, Arab, West Asian, Southeast Asian, Latin American, Japanese and Korean.)

There were one hundred and sixty-three responses to this question. The majority of respondents do not belong to a visible minority group (N=140, 85.89%) and twenty-one did (12.88%). Two people (1.23%) did not wish to respond.

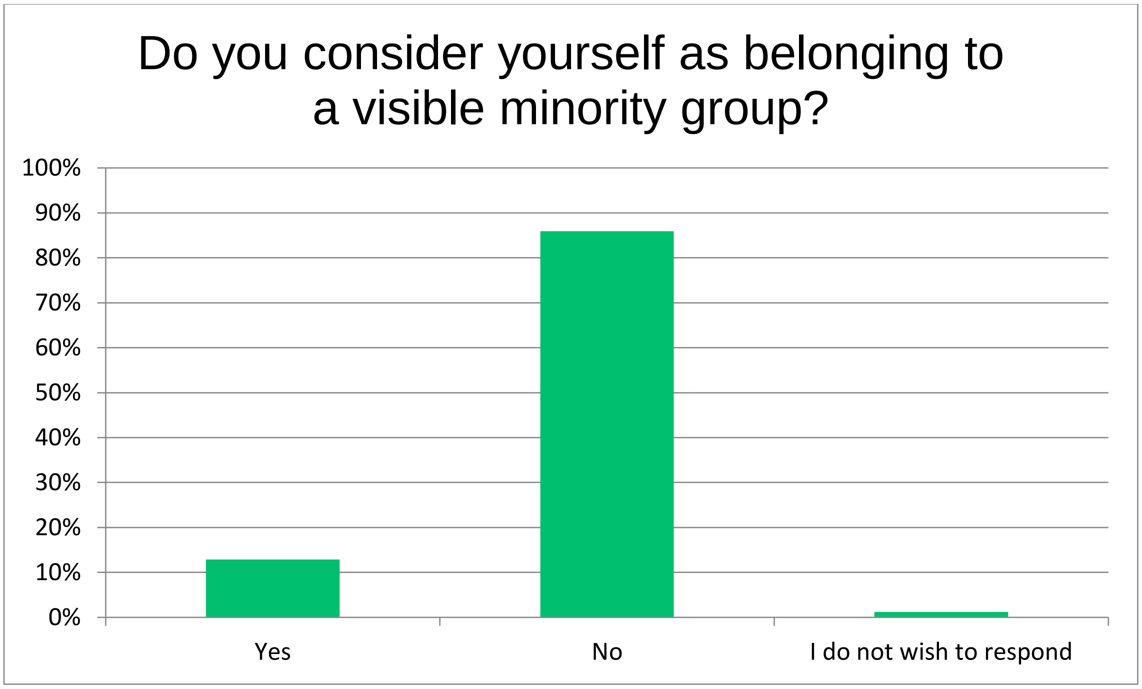

Answer Choices	Percentages	Number
Yes	12.88%	21
No	85.89%	140
I do not wish to respond	1.23%	2

Question 16: Do you consider yourself to be of Indigenous ancestry? (Definition: “Indigenous ancestry” refers to whether a person has ancestry associated with First Nations, Métis, and/or Inuit)

There were one hundred and sixty-three answers to this question. One hundred and fifty-seven (N=96.32%) answered no and 5 answered yes (3.07%). One person did not wish to respond (0.61%).

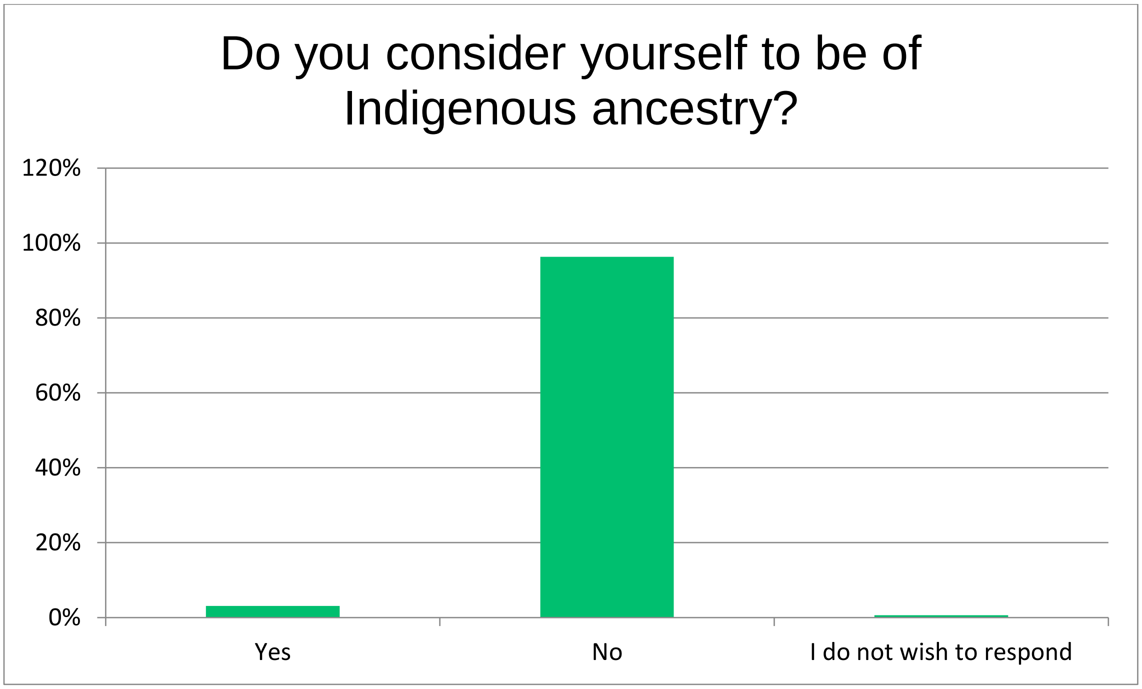

Answer Choices	Percentages	Number
Yes	3.07%	5
No	96.32%	157
I do not wish to respond	0.61%	1
	Answered	163
	Skipped	4

Question 17 : Do you consider yourself to be a person with a disability? Select all that apply. (Note: the social model of disability recognizes that disability is not created by any particular medical or physical condition, but rather by societal barriers.)

There were one hundred and sixty-one responses to this question. One hundred and sixteen (72.05%) do not consider themselves a person of disability. The most common disability is Mental Health-Related (N=32, 19.88%); Cognitive (N=7, 4.35%); sensory (N=2, 1.24%); Other/Unknown (N=4, 2.48%); 4 (N=2.48%) did not wish to respond.

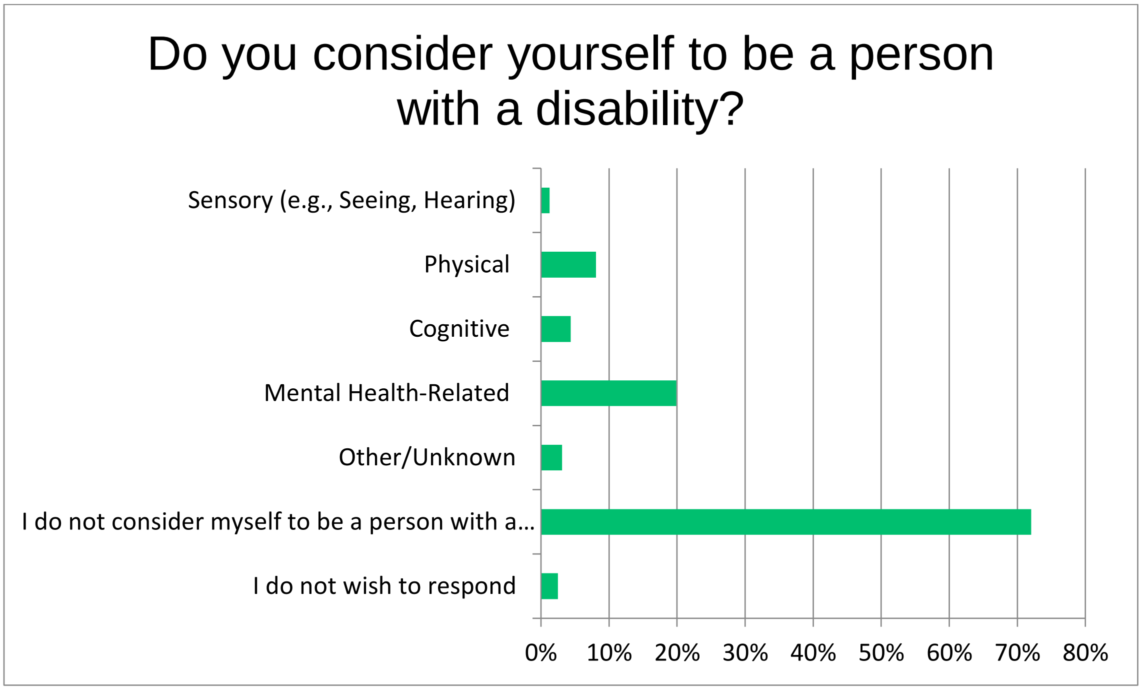

Answer Choices	Percentages	Number
Sensory (e.g., Seeing, Hearing)	1.24%	2
Physical (e.g., Mobility, Flexibility, Dexterity, Chronic Pain)	8.07%	13
Cognitive (e.g., Learning, Developmental, Memory)	4.35%	7
Mental Health-Related (e.g., Depression, Anxiety, ADHD)	19.88%	32
Other/Unknown	3.11%	5
I do not consider myself to be a person with a disability	72.05%	116
I do not wish to respond	2.48%	4
	Answered	161
	Skipped	6

Question 18: Do you identify as 2SLBGTQIA+?(Definition: Two-Spirit, Lesbian, Gay, Bisexual, Transgender, Queer or Questioning, Intersex, Asexual, and additional sexual orientations and gender identities)

There were one hundred and sixty-three answers to this question. The majority do not identify as 2SLBGTQIA+ (N=128, 78.53%); 33 do (20.25%) and 2 (N=1.23%) did not wish to respond.

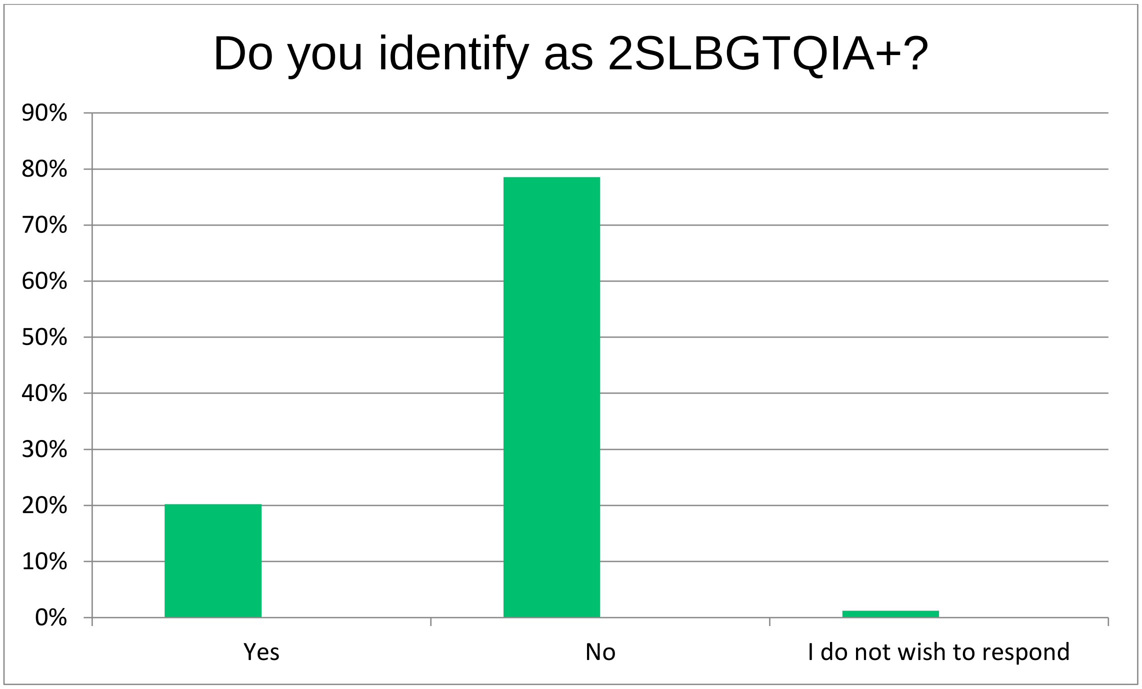

Answer Choices	Percentages	Number
Yes	20.25%	33
No	78.53%	128
I do not wish to respond	1.23%	2
	Answered	163
	Skipped	4

Question 19: What is your age group?

The most popular age range of our respondents (N=162) was in the 35-44 years old range (N=48, 29.63%); followed by 45-54 years (N=47, 29.01%); 25-34 years (N=32, 19.75%); 55-64 years (N=30, 18.52%), and five (3.09%) were in the over 65 years of age range. There were no respondents under 25 years old.

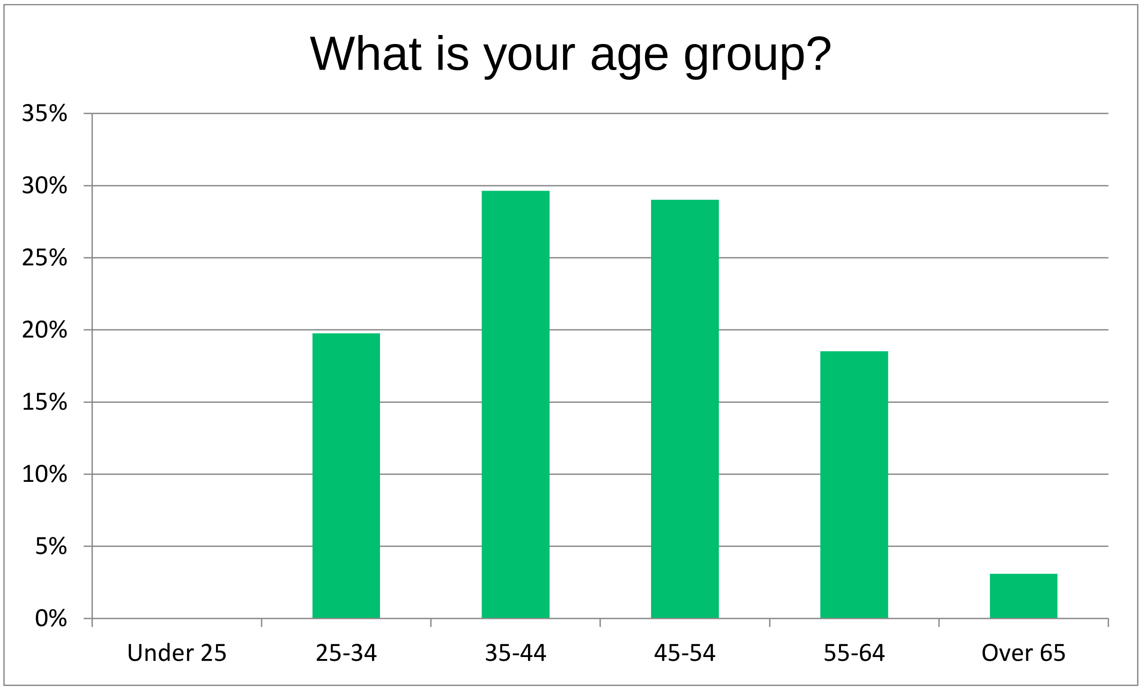

Answer Choices	Percentages	Number
Under 25	0%	0
25-34	19.75%	32
35-44	29.63%	48
45-54	29.01%	47
55-64	18.52%	30
Over 65	3.09%	5

	Answered	162
	Skipped	5

Question 20: Where do you work?

Most of our respondents are from Ontario (N=70, 42.94%); followed by Quebec (N=22, 13.5%); British Columbia (N=19, 11.66%); Alberta (N=13, 7.98%), Saskatchewan (N=13, 7.98%); Nova Scotia (N=11, 6.75%); New Brunswick (N=6, 3.68%); Newfoundland and Labrador (N=2, 1.23%); three responded “other” and specified: remotely, retired and based in Vancouver but servicing several locations online. There is no representation from Prince Edward Island and the territories Northwest Territories, Nunavut, and Yukon.

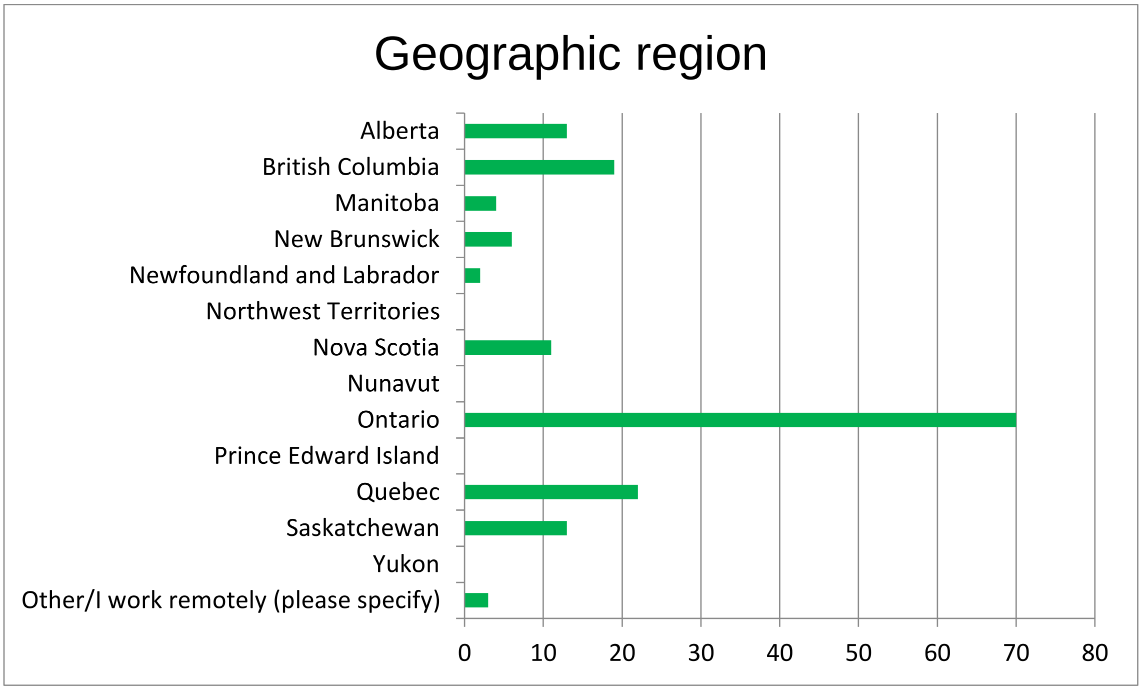

Answer Choices	Percentages	Number
Alberta	7.98%	13
British Columbia	11.66%	19
Manitoba	2.45%	4

New Brunswick	3.68%	6
Newfoundland and Labrador	1.23%	2
Northwest Territories	0.00%	0
Nova Scotia	6.75%	11
Nunavut	0.00%	0
Ontario	42.94%	70
Prince Edward Island	0.00%	0
Quebec	13.50%	22
Saskatchewan	7.98%	13
Yukon	0.00%	0
Other/I work remotely (please specify)	1.84%	3
	Answered	163
	Skipped	4

Question 21: How many years of work experience in health-related libraries or as an information professional in the health industry do you have?

There were one hundred and sixty-three answers to this question. The majority have more than 15 years experience in health-related libraries or as an information professional in the health industry (N=52, 31.90%); followed by the ranges 11-15 years of experience (N=38, 23.31%); 6-10 years (N=35, 21.47%); 1-5 years (N=32, 19.63%); and less than one year (N=6, 3.68%).

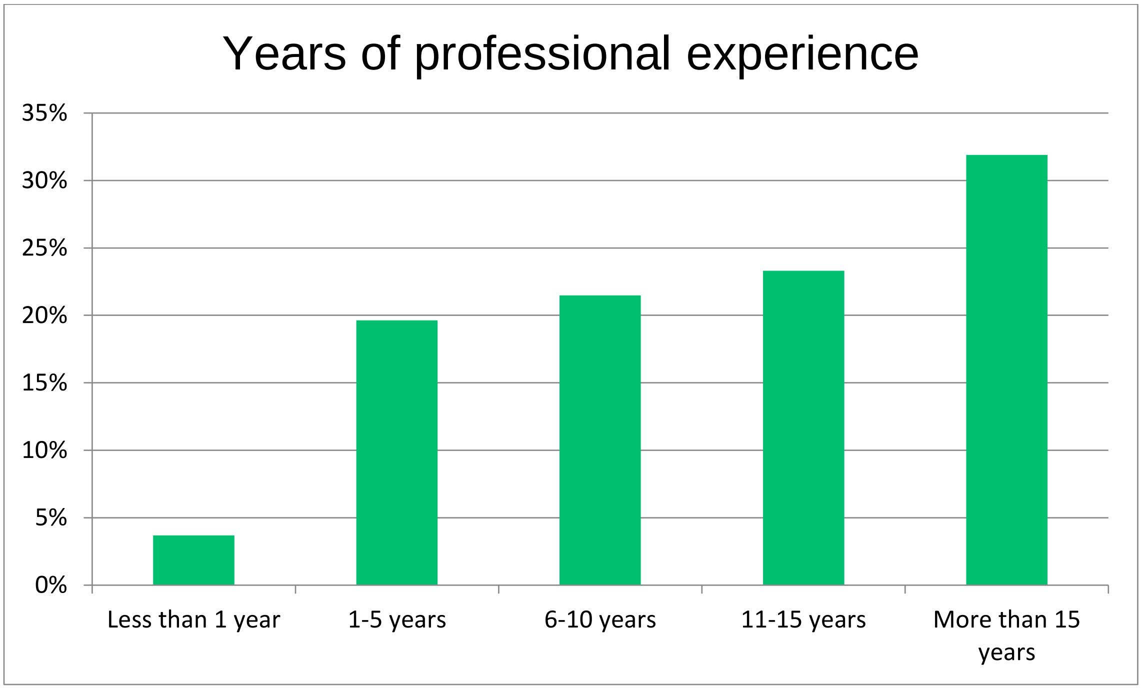

Ranges of years of experience	Percentages	Number
Less than 1 year	3.68%	6
1-5 years	19.63%	32
6-10 years	21.47%	35
11-15 years	23.31%	38
More than 15 years	31.90%	52
	Answered	163

	Skipped	4

Question 22: What is the highest level of formal education in library/information science you have or are currently completing?

There were one hundred and sixty-two answers to this question. The majority (N=142, 87.65%) have a Master's Degree; followed by College/University Diploma (N=11, 6.79%); and a Doctorate/PhD (N=4, 2.47%); a certificate (N=1, 0.62%) and four said "other", listing other degrees equivalent to Masters of Library Science, various Bachelors, one person said "currently enrolled in a PhD" and another person said "no formal degrees".

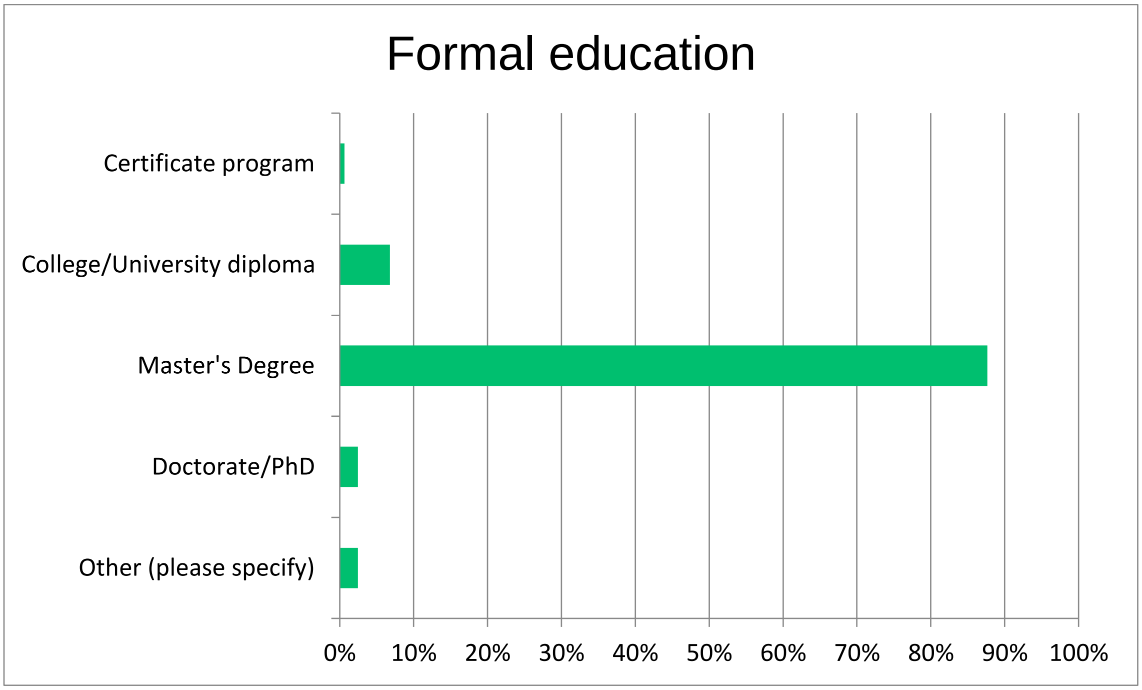

Answer Choices	Percentages	Number
Certificate program	0.62%	1
College/University diploma	6.79%	11
Master's Degree	87.65%	142
Doctorate/PhD	2.47%	4

Other (please specify)	2.47%	4
	Answered	162
	Skipped	5

Question 23: As of January 2023, what is your gross annual salary (before taxes and other deductions)? [Amounts are in Canadian dollars. International respondents please adjust accordingly.]

There were one hundred and sixty-two answers to this question. The majority reported their annual salary in the range of $65,000 to $79,999 (N=35, 21.60%); followed by the range "$110,000 or higher" N=22 (13.58%); "$90,000 to $99,999" (N=22 13.58%); $80,000 to $89,999 (N=19.14%); "under $49,999" (N=9, 5.56%); "$50,000 to $64,999" (N=9, 5.56%). Four said they were not currently earning and seventeen (N=10.49%) did not wish to respond. Five people skipped this question.

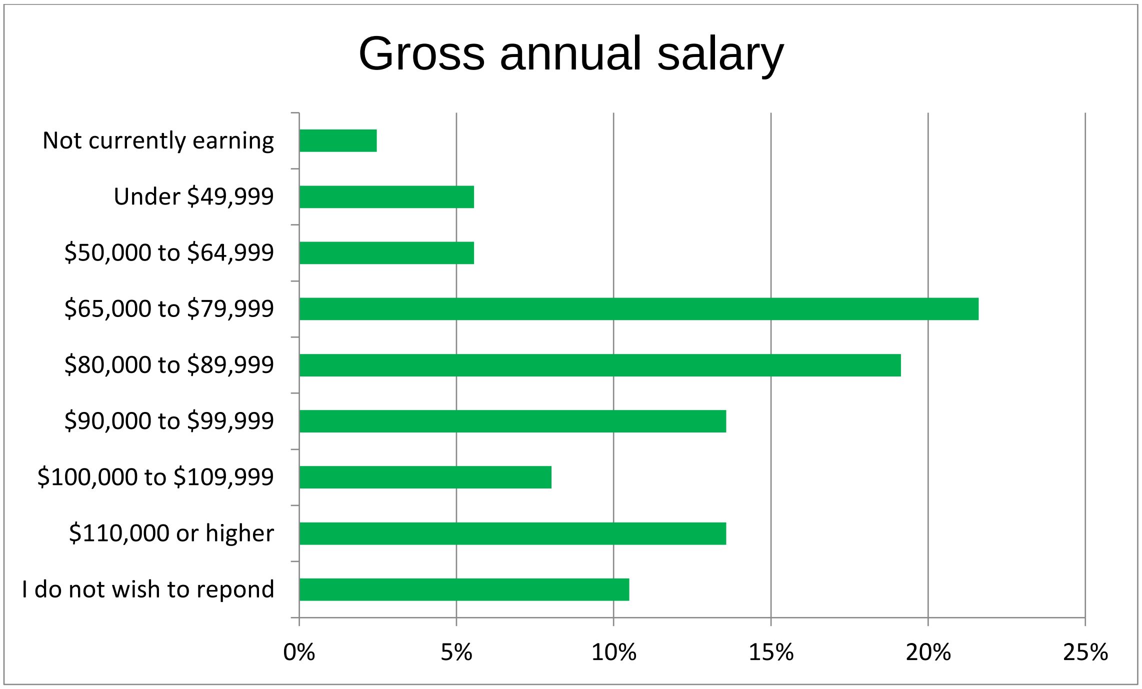

Answer Choices	Percentages	Number
Not currently earning	2.47%	4
Under $49,999	5.56%	9

$50,000 to $64,999	5.56%	9
$65,000 to $79,999	21.60%	35
$80,000 to $89,999	19.14%	31
$90,000 to $99,999	13.58%	22
$100,000 to $109,999	8.02%	13
$110,000 or higher	13.58%	22
I do not wish to respond	10.49%	17
	Answered	162
	Skipped	5

Question 24: What do you think CHLA/ABSC's role should be in regards to EDI?

There were one hundred and sixty-one answers to this question. The majority said CE (N=140, 86.96%); followed by Conference offerings of keynote speakers, special events, etc. (N=137, 85.09%); Grants/Scholarship (N=100, 62.11%); Resource Sharing (N=97, 60.25%); and fifteen said other, which included variations of advocacy, allyship workshops and support, mentoring, mandatory training for board members and conference planners, support for changing hiring practices, management, etc.

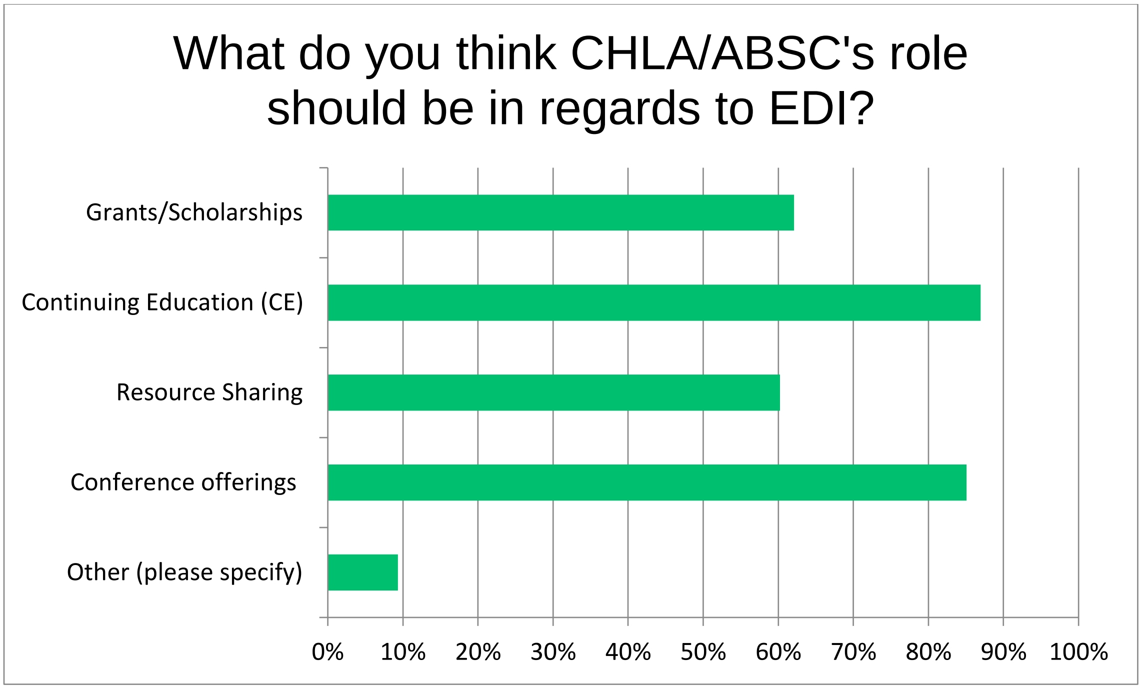

Answer Choices	Percentages	Number
Grants/Scholarships	62.11%	100
Continuing Education (CE)	86.96%	140
Resource Sharing	60.25%	97
Conference offerings (e.g., keynote speakers, special events)	85.09%	137
Other (please specify)	9.32%	15
	Answered	161
	Skipped	6

Question 25: Would you like CHLA/ABSC to offer more CE sessions related to EDI?

There were one hundred and sixty-two answers to this question (N=162). The majority said yes (N=140, 86.42%) and twenty-two said no (13.58%). Five people skipped this question.

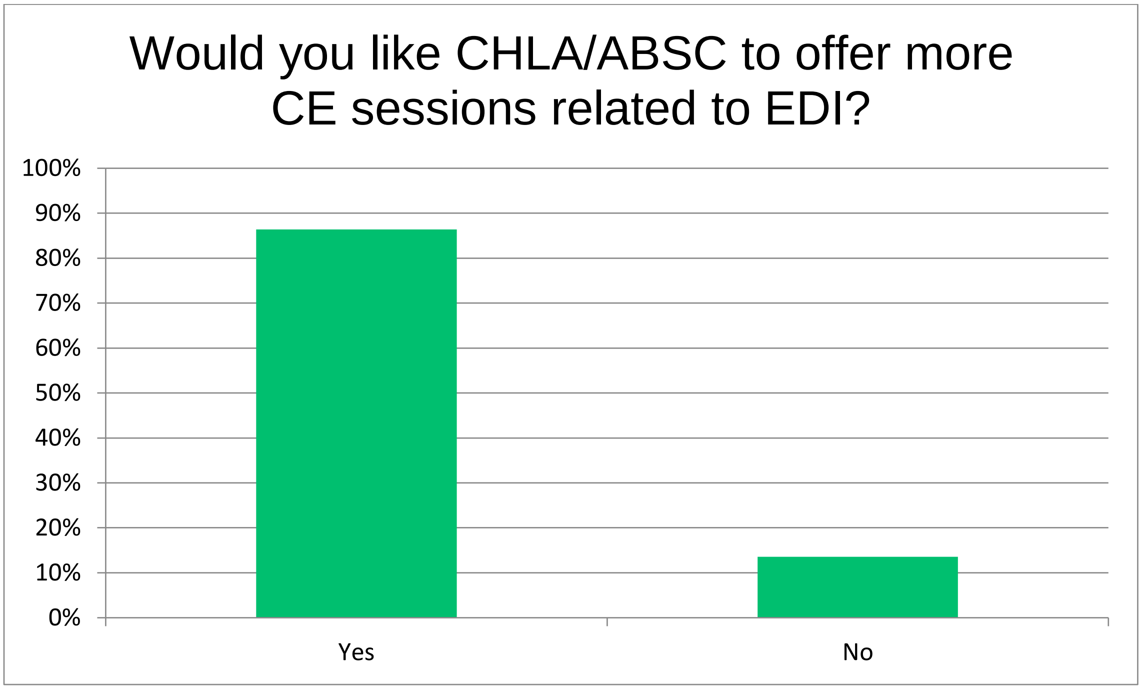

Answer Choices	Percentages	Number
Yes	86.42%	140
No	13.58%	22
	Answered	162
	Skipped	5

Skip to question 27 if "Yes" is selected.

Question 26: Why not?

Twenty-two respondents (13.58%) said CHLA/ABSC should not provide more CE related to EDI, and provided feedback for their choice. Eleven (50%) said "I would prefer CHLA/ABSC CE focus on professional skills", and five (22.73%) said "I already receive EDI training via my institution".

Six answered "other", specifying that they felt EDI was too easy to ignore and expressing doubts about whether CE sessions were the right approach to address EDI issues within the association. Respondents also expressed that what had been offered thus far had duplicated training already received via their institutions, and reiterated that CHLA/ABSC CE offerings should focus on the development of professional competencies.

Question 27: Please specify which EDI related topics would most interest you in a CHLA/ABSC CE session?

This was an open-ended question and seventy-seven responses were received. The following are themes that emerged from the responses: allyship (N=13); Indigenous knowledge, resources, guest speakers or related topics (N=10); Improving collections such as decolonization of collections and improving subject headings (N=10); Instruction with an EDI lens (N=6); Improving the website and/or conference for members with non-visible barriers/disabilities (N=4); Speakers against anti-Semitism (N=2); and women in executive positions in libraries (N=1).

Question 28: Please reflect back on your most recent CHLA/ABSC experiences and respond to the following statements

One hundred and fifty-three people responded to this question, while fourteen skipped it.

	Strongly agree N (%)	Agree N (%)	Neither agree nor disagree N (%)	Disagree N (%)	Strongly disagree N (%)	Total	Weighted Average
I have felt welcome and included at CHLA/ABSC events	(54) 35.29%	(66) 43.14%	(32) 20.92%	(1) 0.65%	0	153	4.13
I feel I am treated with respect at CHLA/ABSC events	(66) 43.14%	(63) 41.18%	(23) 15.03%	(1) 0.65%	0	153	4.27
There are opportunities within CHLA/ABSC for me to participate at a level I feel comfortable with	(49) 32.03%	(77) 50.33%	(24) 15.69%	(3) 1.96%	0	153	4.12
I feel comfortable sharing my personal perspective and experiences within CHLA/ABSC	(40) 26.14%	(59) 38.56%	(44) 28.76%	(9) 5.88%	(1) 0.68%	153	3.84
						Answered	153
						Skipped	14

Question 29: Based on your answers above, what do you think CHLA/ABSC could do to improve?

There were a total of sixty-three responses to this question, while one hundred and four people skipped this question. The most common suggestions among respondents were about providing CE or workshops or inviting guest speakers participating in events such as human libraries, conference keynotes and presentations; making the annual conference and other events more accessible to members; and making opportunities for everyone to participate in conferences, on the Board, and in the profession in general. There were a number of responses where members expressed that they were too new to the association or the profession, and so they felt that they could not comment on EDI. At the same time, other respondents felt that their comments should be given less weight because they represent the majority of the membership (white, female), and that the suggestions of visible minority members should be prioritized.

Question 30: Based on your answers above, what do you think CHLA/ABSC should continue doing?

Fifty people answered this question, and one hundred and seventeen skipped this question. Respondents encouraged CHLA/ABSC to continue engaging members in the EDI conversation, providing networking and professional development opportunities, ensuring the conference is welcoming and accessible, and having diverse guest speakers at conferences and workshops. Some expressed the importance of free or low-cost online events so that they are accessible to all.

Question 31: Is there anything else you would like to share?

Thirty-four people answered this question. Most of the responses expressed gratitude for the opportunity to provide feedback. However, other answers included comments and suggestions on the survey design/cautions on interpretations of results. There were requests for CHLA/ABSC to create concrete plans, actions and accountability for EDI. A link to the MLA climate survey was also included.
